# Different fatty acid patterns in serum in patients with various thyroid diseases

**DOI:** 10.1038/s41598-025-33974-9

**Published:** 2026-01-05

**Authors:** Martyna Lukasiewicz, Agata Zwara, Monika Czapiewska, Natalia Koc, Jan Opalko, Agata Janczy, Adriana Mika, Andrzej Hellmann

**Affiliations:** 1 Department of Surgical Oncology, Transplant Surgery and General Surgery, Medical University of Gdansk, Smoluchowskiego 17, 80-214 Gdansk, Poland; 2 Department of Otorhinolaryngology, Head and Neck Surgery, Medical University of Warsaw, Banacha 1a, 02-097 Warsaw, Poland; 3 Department of Environmental Analytics, Faculty of Chemistry, University of Gdansk, Wita Stwosza 63, 80-308 Gdansk, Poland; 4 Department of Pharmaceutical Biochemistry, Medical University of Gdansk, 1 Debinki, 80-211 Gdansk, Poland; 5 Department of Food Commodity Science, Medical University of Gdansk, ul. Debinki 7, 80-211 Gdansk, Poland

**Keywords:** Fatty acids, Thyroid, Inflammation, Biomarker, Early diagnosis, Lipids, Predictive markers, Endocrine system and metabolic diseases

## Abstract

Thyroid diseases and obesity are major health issues in the 21st century. While fatty acids (FAs) are known to influence various conditions, their role in thyroid disorders remains unclear. This study investigated FA profiles in patients with thyroid diseases to assess their diagnostic potential. From 891 patients treated at the Thyroid Cancer Center of the Medical University of Gdansk (2021–2024), 173 were selected for FA analysis using gas chromatography-mass spectrometry. Receiver operating characteristic (ROC) analysis evaluated the predictive value of individual FAs, and metabolite set enrichment analysis (MSEA) identified changes in FA metabolism. Results showed significant alterations in FA profiles among thyroid disease patients. Notably, levels of specific n-3 polyunsaturated FAs (ALA, EPA, DHA) were reduced in Hashimoto’s thyroiditis (HT) compared to healthy controls, despite no significant change in total PUFA levels. ROC analysis highlighted certain FAs with high AUC values, especially in HT and follicular cell adenoma, indicating potential as diagnostic biomarkers. MSEA revealed disruptions in pathways related to α-linolenic and linoleic acid metabolism and β-oxidation of very long-chain FAs. These findings suggest FA profiling could support the diagnosis of thyroid disorders and offer new insights into disease-related metabolic changes.

**Supplementary Information:**

The online version contains supplementary material available at 10.1038/s41598-025-33974-9.

## Introduction

Thyroid diseases are a significant global health problem, with incidence rates steadily increasing worldwide^1^. They result from a complex interplay of genetic, environmental, and immune factors. Among them, papillary thyroid cancer (PTC), follicular-cell adenoma (FCA), nodular goiter (NG), and Hashimoto’s thyroiditis (HT) exhibit distinct etiological patterns. PTC, the most common thyroid malignancy, is strongly linked to childhood radiation exposure^2^, and is frequently linked to iodine excess^3,4^, while FCA, has a weak correlation with iodine imbalance, may arise from radiation and carries a risk of transformation into follicular carcinoma^5^. In turn, NG, typically caused by iodine deficiency, leads to compensatory glandular hyperplasia^3^, whereas HT, an autoimmune disorder, results in T-cell-mediated thyroid destruction and hypothyroidism^6^ and excessive iodine intake may exacerbate HT^5,7^. Autoimmunity is a defining feature of HT but is absent in FCA and NG^6,8,9^. What is more, PTC is not inherently autoimmune, though coexisting autoimmune conditions may increase its risk^10^. Therefore, thyroid autoantibodies are a great tool in HT diagnosis, which is missing for other thyroid diseases.

Altered lipid metabolism is associated with numerous diseases. In recent years, increasing attention has been given to lipidomics as a promising tool for studying thyroid diseases, with the potential to enhance diagnostic and therapeutic strategies. Elevated fatty acids (FAs), a key feature of metabolic syndrome, contribute to insulin resistance, inflammation, and dyslipidemia, which may further influence thyroid hormone function and metabolism. Emerging scientific evidence suggests significant alterations in serum FA profiles across various thyroid disorders^11^. FAs are essential metabolites that not only serve as a major energy source but also play critical roles in inflammation, cellular signalling, and metabolic regulation. Their involvement in these fundamental biological processes underscores their potential as biomarkers for thyroid dysfunction^12^. The intense β-oxidation of very long-chain FAs (VLCFAs) is associated with the inflammatory process^13^, and the progression of PTC^14^. Also, medium-chain FAs (MCFAs) are involved in generation of the inflammation^15^.

In this study we wanted to answer the question how thyroid gland diseases affect the FAs profile in serum and if FAs changes in the serum of patients are specific for different thyroid diseases. We also wanted to find out whether we can determine the entity of thyroid disease based on the FAs panel or a single FA and whether this can become a new diagnostic strategy.

## Materials and methods

### Patients

The present study included patients who underwent thyroidectomy or lobectomy at the Thyroid Cancer Center of the Medical University of Gdansk between January 2021, and December 2024. The study cohort was divided into the following groups: 51 patients with NG, 51 with PTC, 33 with FCA, and 38 with HT, who did not undergo surgery (Fig. S1). A healthy control (HC) group comprised 31 individuals without thyroid disease. Extensive medical histories were obtained from all participants, including information on hypertension, chronic kidney disease, heart failure, ischemic heart disease, cerebrovascular conditions, dyslipidemia, insulin resistance, type 2 diabetes mellitus, thyroid diseases, allergies, and current medications. Written informed consent was obtained from all study participants prior to inclusion. Clinical data were collected from medical records. Pathologic diagnoses were made according to the World Health Organization criteria. Routine laboratory parameters were assessed at the Central Clinical Laboratory of the Medical University of Gdansk (Supplementary Table 1). The diagnosis of HT was confirmed based on elevated levels of thyroglobulin antibodies (TgAbs) and thyroid peroxidase antibodies (TPOAbs), along with characteristic ultrasound findings. In all study groups, participants were women. Blood samples were collected in the morning after overnight fasting from all study participants. Following centrifugation, serum samples were aliquoted and stored at -80 °C to preserve their integrity for subsequent analyses.

### Ethical statement

The present study was approved by the Independent Bioethics Committee for Scientific Research at the Medical College of Gdansk, where it was conducted, under the number NKBBN/62/2021.

The study was performed in agreement with the Declaration of Helsinki of the World Medical Association.

### GC-MS analysis of fatty acids

Total lipids were extracted from whole serum samples using a chloroform-methanol mixture (2:1, v/v). 300 µL of serum was used to the FAs analysis. The total lipid extracts were hydrolyzed with 1 mL of 0.5 M KOH in methanol at 90 °C for 3 h. FA methyl esters (FAMEs) were prepared with 1mL of a 10% boron fluoride in methanol solution and then heated at 55 °C for 90 min. After that 1mL of water was added to the reaction mixture, the FAMEs were extracted three times with 1mL of n-hexane, and the solvent was evaporated. After derivatisation, FA methyl esters were analyzed by gas chromatography-electron ionization mass spectrometry QP-2020 NX (Shimadzu, Japan) and separated on a 30 m Zebron ZB-5MSi capillary column with 0.25 mm i.d. (film thickness 0.25 mm) as described previously^16^. An amount of 1µL of the sample was injected at a split mode (ratio 20:0). The temperature of injection, ion source and transfer line were 300 °C, 200 °C and 300 °C, respectively. The column temperature was set in a range of 60–300 °C (4 °C min^− 1^), with helium as the carrier gas at the column head pressure of 60 kPa. The electron energy used for FAME ionization was 70 eV. Full scan mode was employed with a mass scan range of m/z 45 to 700.

### Quantification protocol and data analysis

After extraction, the mass of lipids was weighed, and based on the total volume of serum used for extraction, we calculated the amount of lipids per 1 ml of serum. In order to quantification of total number of FAs 19-methylarachidic acid was used as an internal standard. Accurate identification of the FA profile was achieved using reference standards (BCFA and VLCFA standards, Larodan, and 37 FAME Mix, Sigma-Aldrich). FA concentrations were calculated based on the internal standard signal and expressed as relative abundance. These values were then calculated for a specific serum volume, and the finally, the results has been shown as a µmol of FA/1L of serum. We identified 40 fatty acids and, based on these, defined 11 additional groups to which the acids were classified (all are mentioned in the manuscript and shown in Supplementary Figures). The lowest fatty acid concentration determined was 0.05 µM, corresponding to 0.01% of fatty acids in the mixture of 40 analytes.

Obtained data were analysed with SigmaPlot 14.5 (Systat Software Inc., San Jose, CA, US). All values are expressed as mean ± standard deviation (SD). The Shapiro–Wilk test was used to assess the normality of the data distribution. For quantitative measures, Kruskal–Wallis ANOVA of Variance on Ranks followed by All Pairwise Comparison Dunn’s Method on ranks was used. A value of *p* < 0.05 was considered statistically significant. PCA and Receiver operating characteristic (ROC) analysis was performed in MetaboAnalyst 6.0v^17^ to assess the area under the curve (AUC) and to compare the predictive ability of significant FAs between the tested groups. The raw data was normalized to the total area and autoscaled. The linear SVM algorithm was used to generate the ROC curve. To understand whether it is possible to increase the predictive power, the single ROC curve was constructed for all comparisons using only the FAs with a p-value < 0.01. When analysing the ROC curve for selected sets of FAs, 10-fold Coss validation was used to create a logistic regression model and calculate power. The identified serum FAs were used to investigate the major biological signalling pathways in patients with thyroid disease. In this context, metabolite enrichment pathway analysis (MSEA) was performed using MetaboAnalyst (v6.0), with p-value < 0.05 and FDR < 0.1 considered significant.

## Results

### Biochemical parameters measured in blood of patients: anthropometric and clinical parameters

Only patients with HT differed statistically significantly from the control group by a higher CRP value. In patients with FCA, the CRP value was significantly lower than in patients with PTC and HT (Supplementary Table S1). Interestingly, patients with FCA and PTC had significantly lower TSH values, which is an effect of taking drugs. TSH values in patients with HT were in line with the norms but were significantly higher than in the other study groups.

### Changes in the entire FA panel in serum depending on the disease of thyroid

The principal component analysis results did not reveal a significant difference in the serum FA profiles among patients with thyroid diseases and healthy groups. The majority of variability in the dataset (PC1, 21.23%) was associated with a broad dispersion of the results within all five groups (Supplementary Fig. S2).

**Fig. 1:**
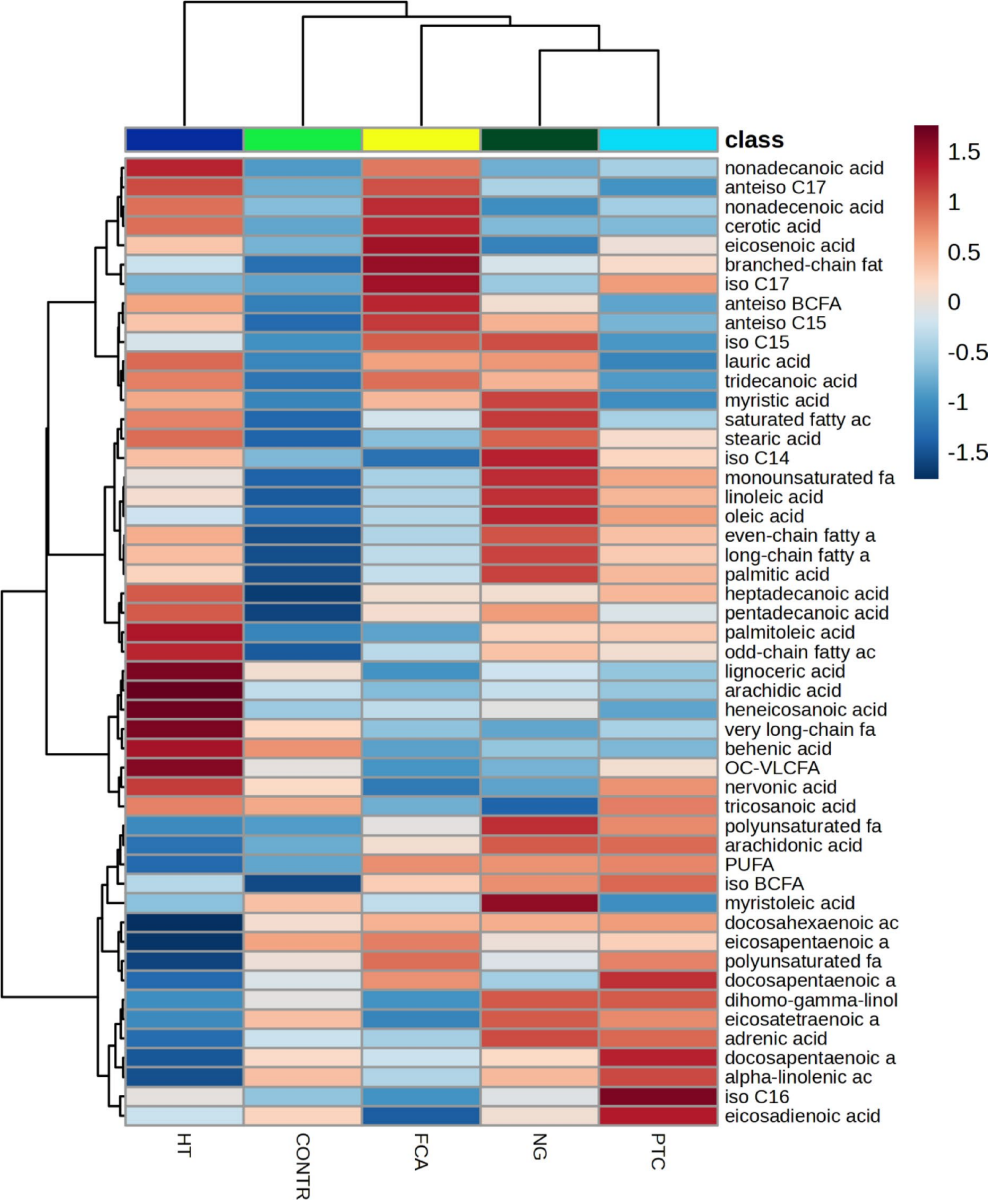
Heatmap visualization of serum fatty acids (FAs) with hierarchical clustering analysis (HCA). Average concentration was used to indicate the level of difference for single FAs among five research groups.

Heatmap representation of the serum FA dataset with hierarchical clustering analysis (HCA) was performed for a graphical representation of FAs that were significantly altered in the different groups (Fig. 1). These data indicate specific patterns of differences in the serum FAs levels between patients with different thyroid diseases and healthy volunteers. In fact, every studied thyroid disease represents specific FA profile different from HC or other studied thyroid diseases. The highest levels of polyunsaturated FAs (PUFAs) were observed in the serum of patients with PTC, while the levels of anteiso branched-chain FAs (BCFAs) were highest in the FCA group (Fig. 1). HT was also characterized by a large amount of anteiso BCFAs, but also of odd-chain FAs (OCFAs) and high levels of VLCFAs. Long-chain FAs (ECFAs) and n6 PUFAs were also elevated in NG. Interestingly, a comparison of the FAs in the serum of patients with 4 studied diseases and in the healthy subjects showed that the lowest levels of some FAs were found in HT and in the healthy subjects (representatives of n3 PUFAs, BCFAs, OCFAs, ECFAs and monounsaturated FAs - MUFAs).

### Selected serum FA as biomarkers of thyroid diseases

**Fig. 2:**
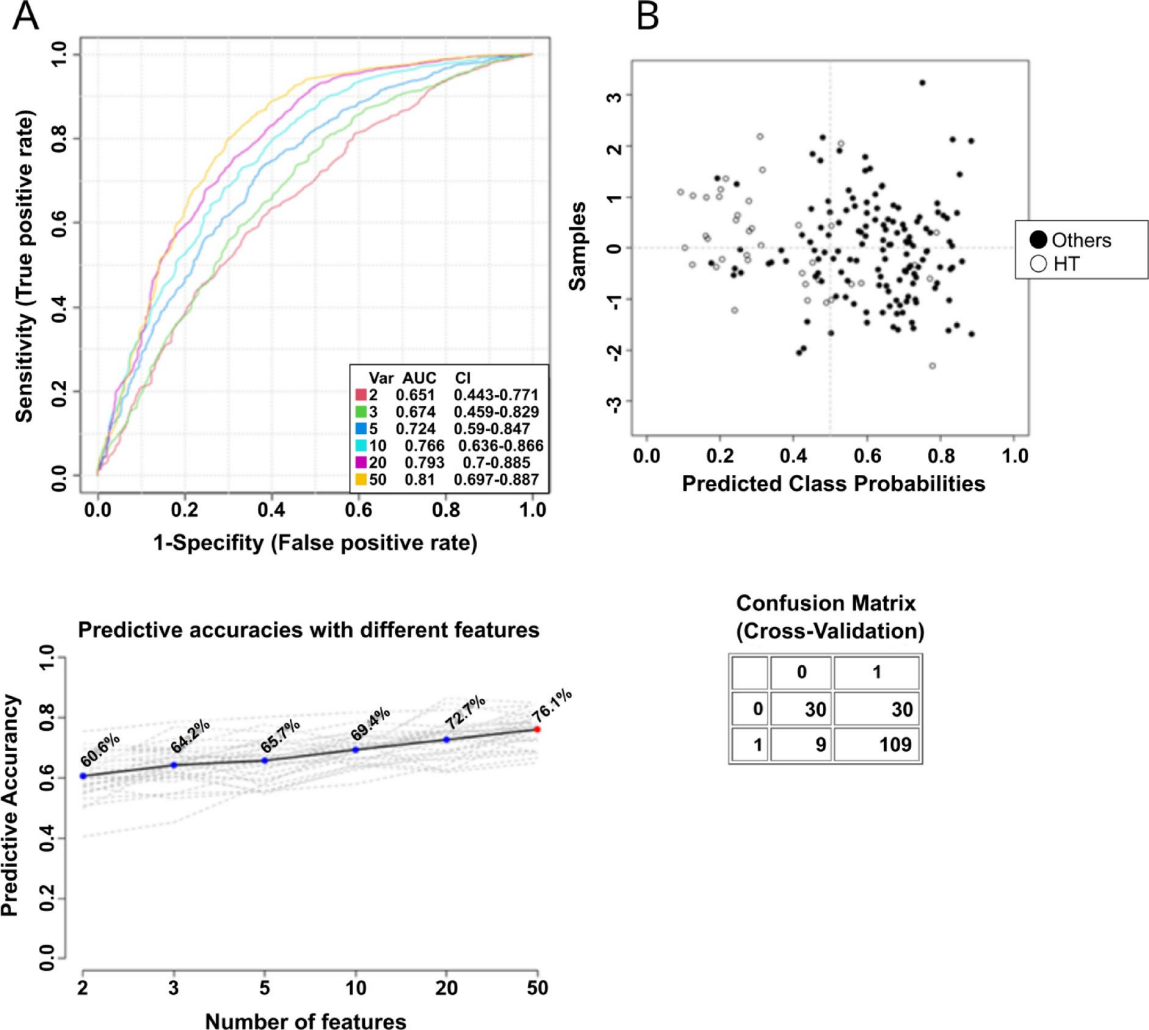
ROC curve of FA indicators from serum predicting the occurrence of HT disease vs. other analyzed groups.

In the next step, serum FAs as a potential marker for thyroid disease were evaluated using the ROC curves. After reviewing all models, the best model (highest AUC) was obtained when the target group was HT. Prediction accuracy with 50 features was 76.1% (Fig. 2A). Figure 2B shows the predicted class probabilities (average of cross-validation) for each sample using the best classifier (based on AUC) by SVM as the classification method.

**Fig. 3:**
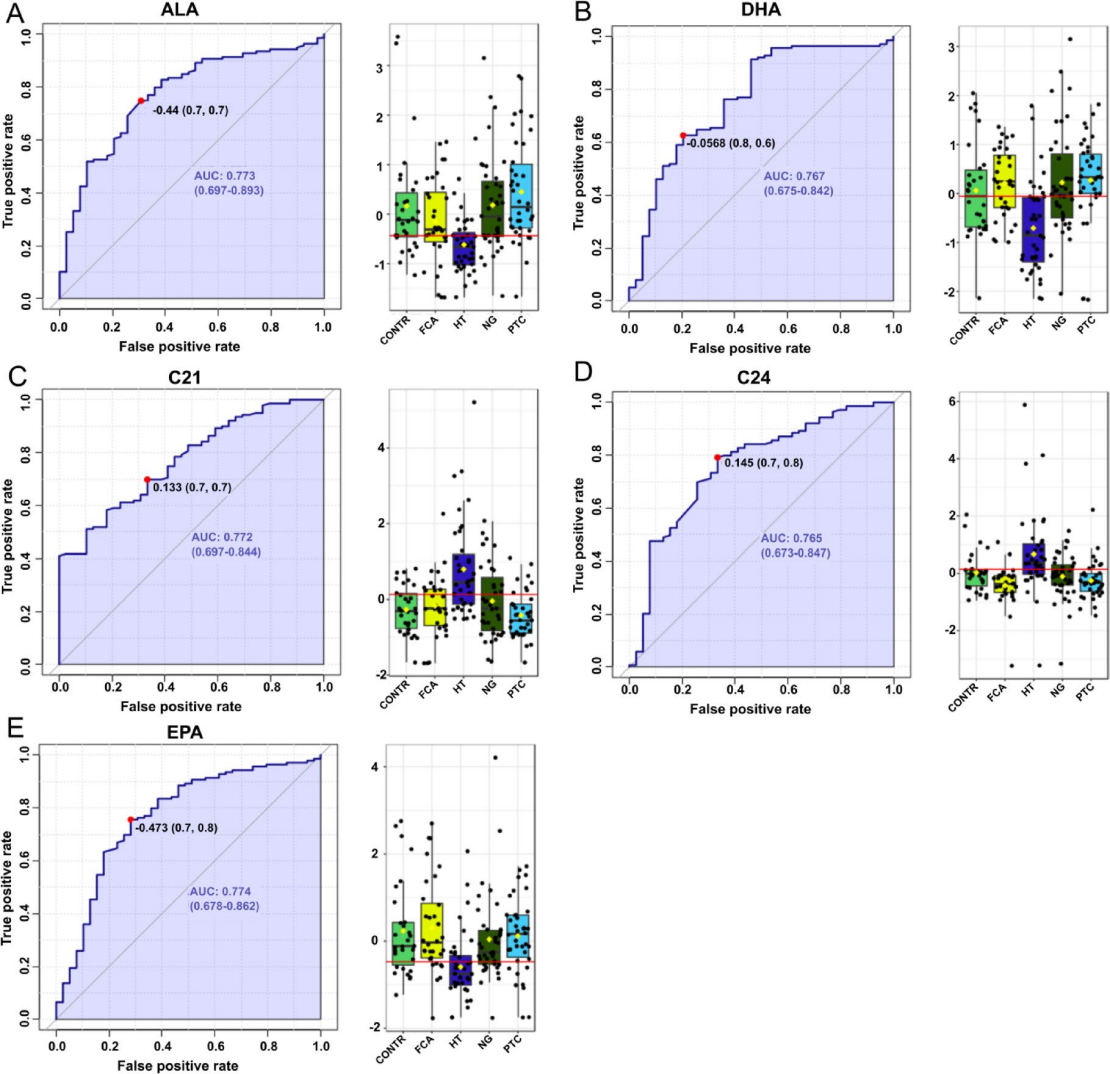
Box plots for the most significantly altered fatty acids for Hashimoto’s thyroiditis, when this disease is a target group. The x-axis shows the specific FA, and the y-axis is the normalized peak intensity. ALA- α-linolenic acid, DHA – docosahexaenoic acid, C21 - heneicosanoic acid, C24 - lignoceric acid, EPA – eicosapentaenoic acid.

In turn, the highest AUC in HT patients’ group was recorded for serum FA including α-linolenic acid (ALA) (0.773), 21:0 (0.772), eicosapentaenoic acid (EPA) (0.774), docosahexaenoic acid (DHA) (0.767) and 24:0 (0.762) (Fig. 3). The other AUC values were included in the Supplementary Table S2. Based on the input FA analysis data for the 5 groups studied, the average accuracy in predicting HT disease based on 100 cross-validations was 0.81 (Fig. S3).

**Fig. 4:**
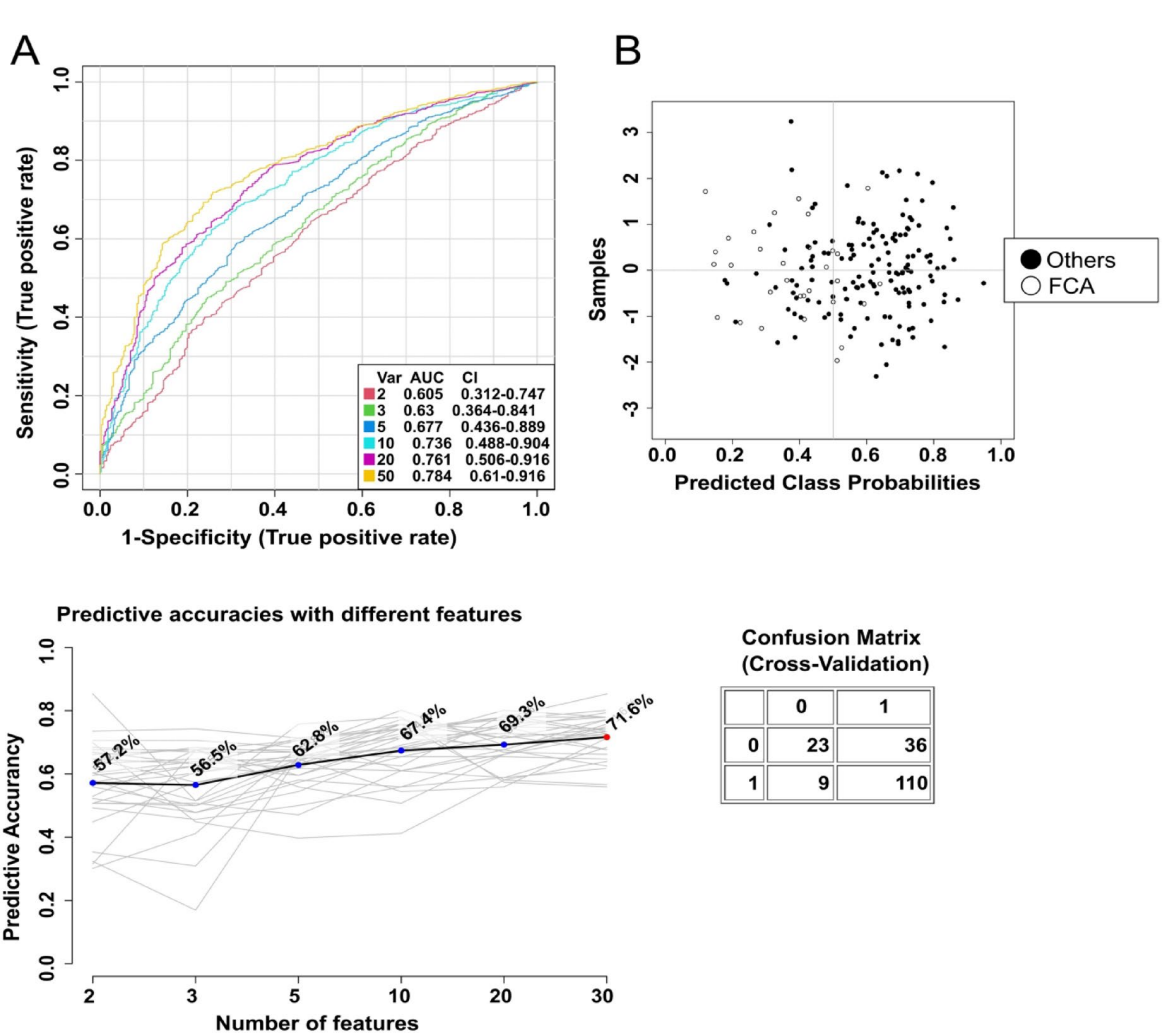
ROC curve of FA indicators from serum predicting the occurrence of follicular-cell adenoma (FCA) vs. other analyzed groups.

The second disease entity with predictive power was FCA. Based on the input FA analysis data the prediction accuracy with 50 FAs was 71.6% (Fig. 4), and the average accuracy in predicting FCA disease based on 100 cross-validations was 0.796 (Fig. S4).

**Fig. 5:**
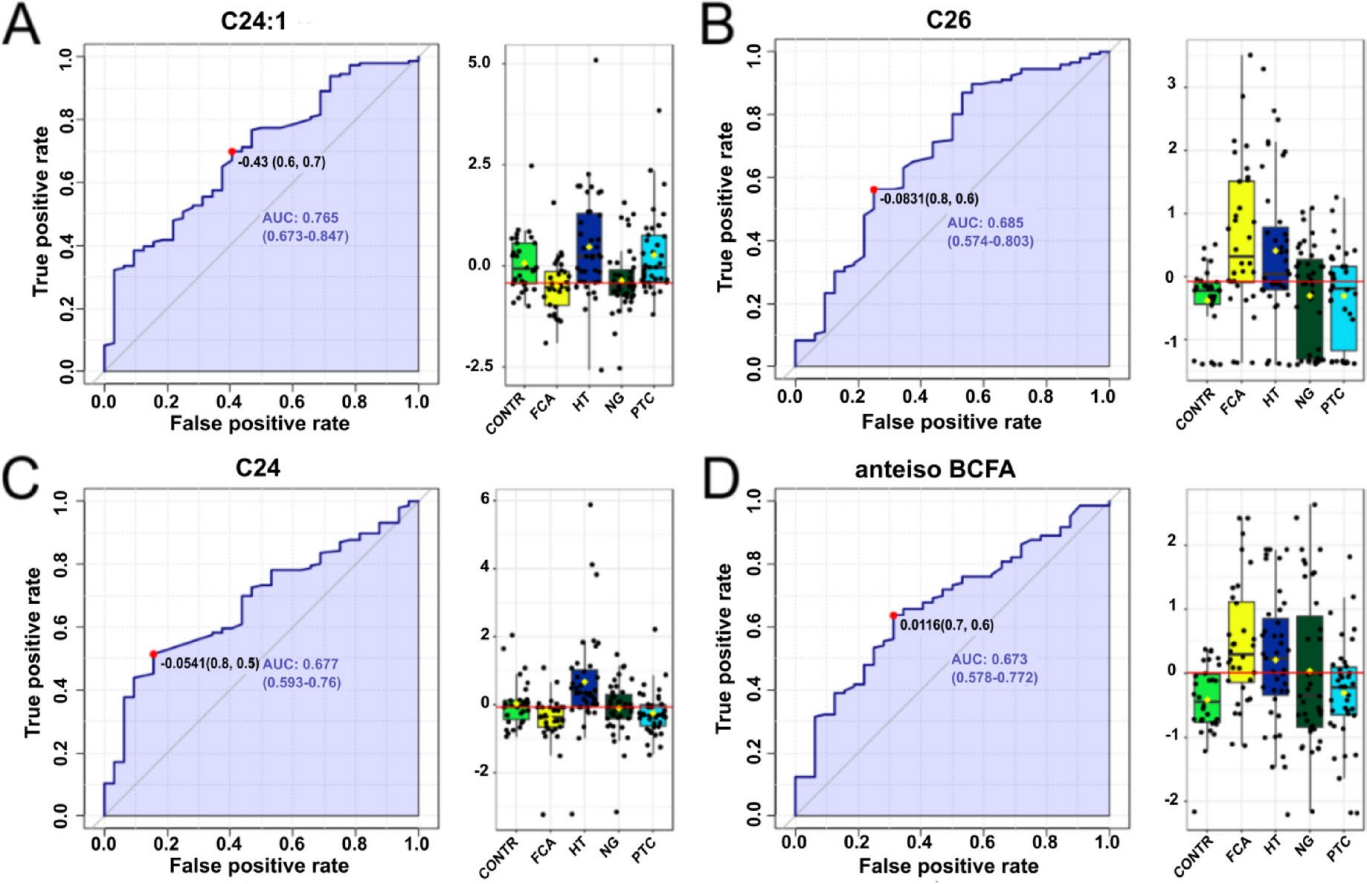
ROC curves for follicular-cell adenoma (FCA) disease and corresponding boxplots of serum content of selected fatty acids (FAs) in different thyroid diseases. C24:1 - nervonic acid, C26 – cerotic acid, C24 – lignoceric acid, anteiso BCFA – anteiso branched-chain fatty acids.

FAs with higher AUC in FCA patients were 24:1 (0.691), 26:0 (0.685), 24:0 (0.677) and anteiso BCFA (0.673) (Fig. 5), and other AUC values were included in the Supplementary Table S3.

**Fig. 6:**
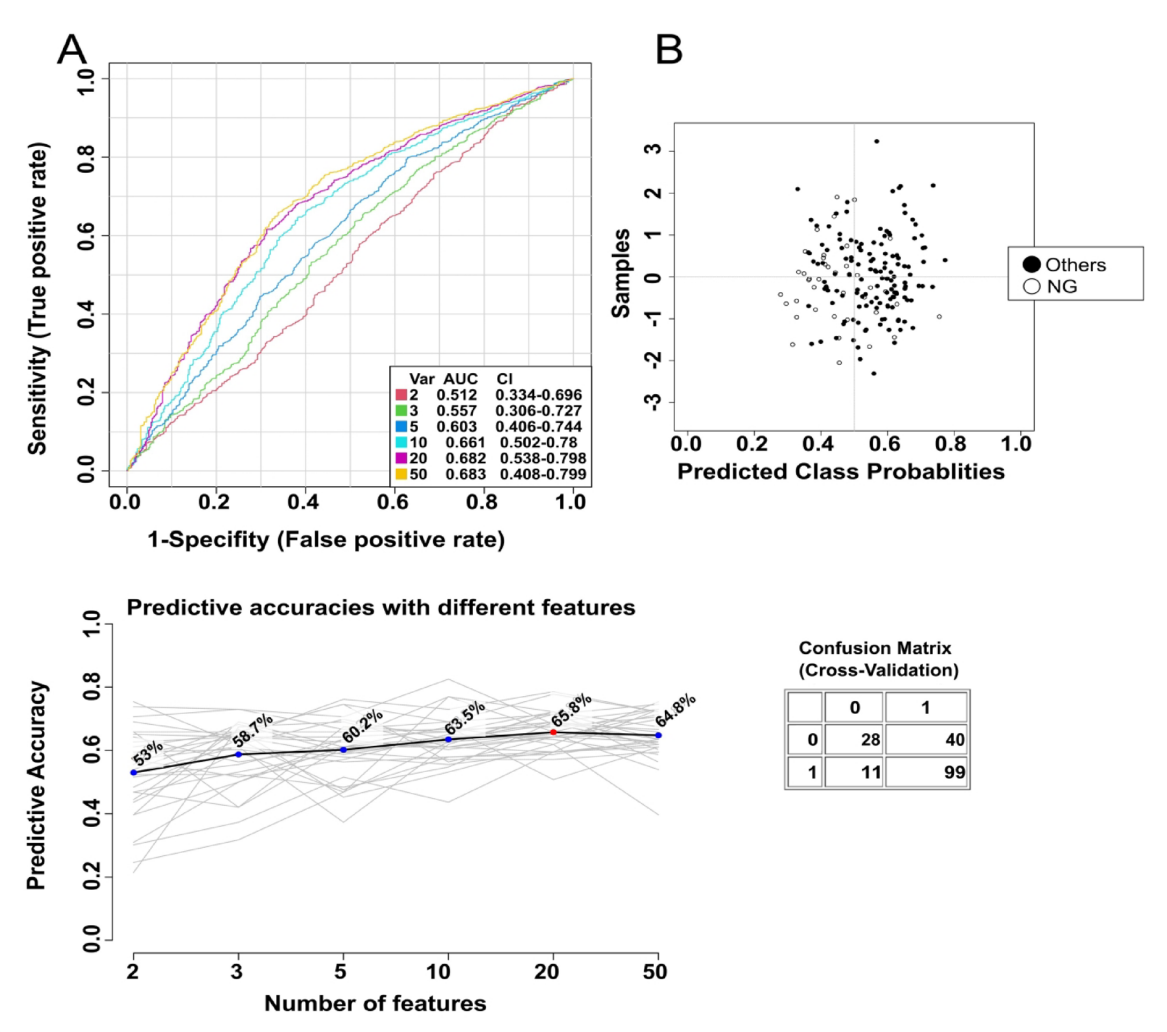
ROC curve of FA indicators from serum predicting the occurrence of nodular goiter (NG) disease.

The predictive power of NG was on the edge of significance. The prediction accuracy with 20 traits was 65.8% (Fig. 6), and the average accuracy in predicting NG disease based on 100 cross-validations was 0.681 (Fig. S5).

**Fig. 7:**
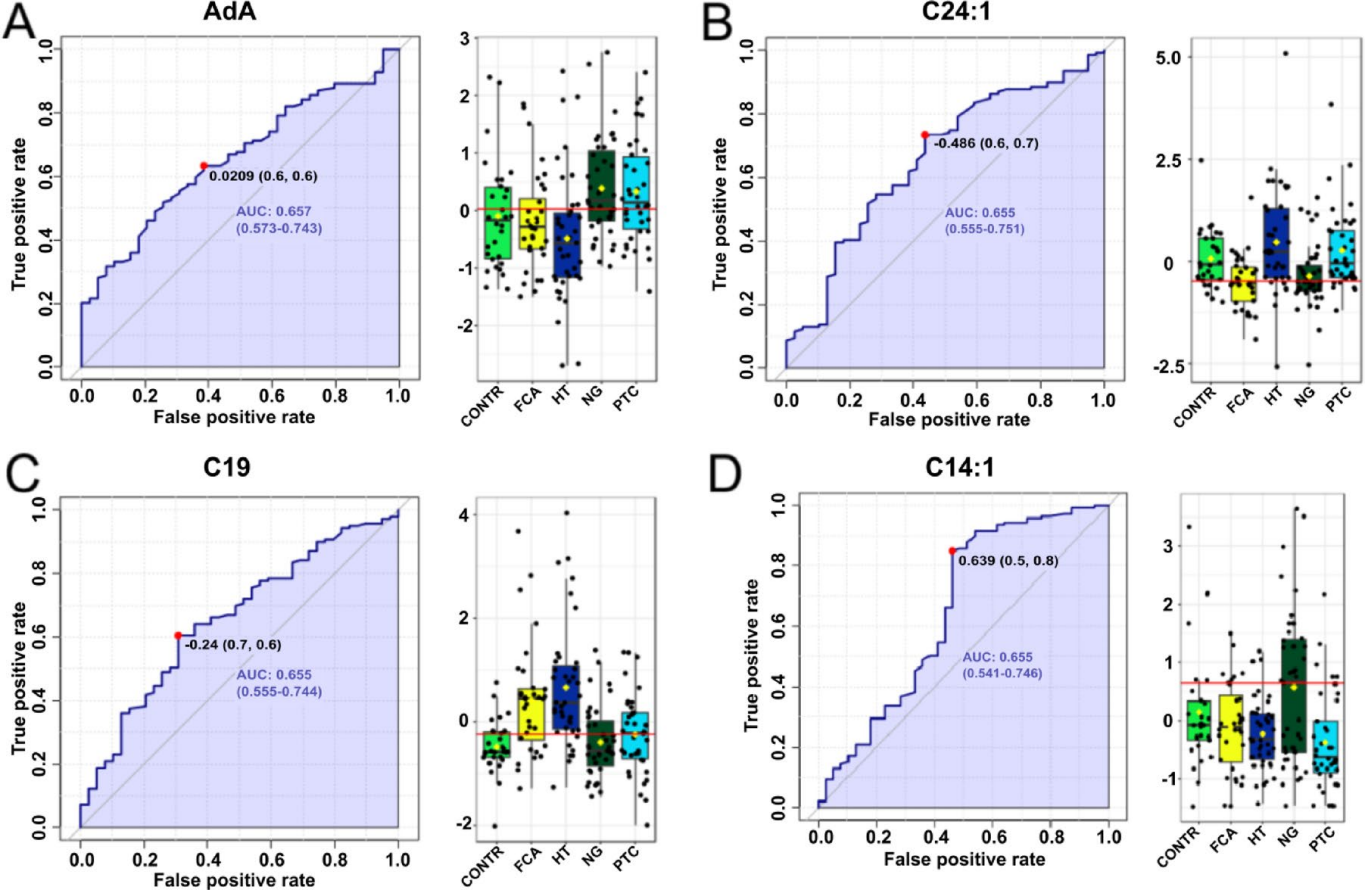
ROC curves for nodular goiter (NG) disease and corresponding boxplots of serum content of selected fatty acids in different thyroid disease groups. AdA – adrenic acid, C24:1 - nervonic acid, C19 – nonadecanoic acid, C14:1 – myristoleic acid.

Similar to other cases, for NG we also determined FAs with the highest AUC value, and they were adrenic acid (AdA) (0.657), 24:1 (0.655), 19:0 (0.655) and C14:1 (0.655), although their values were below the predictive power (Fig. 7, Supplementary Table S4).

Interestingly, the worst scores in the ROC analysis were obtained among the studied groups when PTC was the target group. The prediction accuracy for the different 10 models was 61.8%, and the best model^1^ performed very poorly, indicating that the predictive power of serum FAs for PTC is low (Fig. S6). The average accuracy in predicting PTC disease based on 100 cross-validations was 0.62 (Fig. S7).

### Overview of enriched metabolite sets - biological significance of changes of FA concentration patterns

**Fig. 8:**
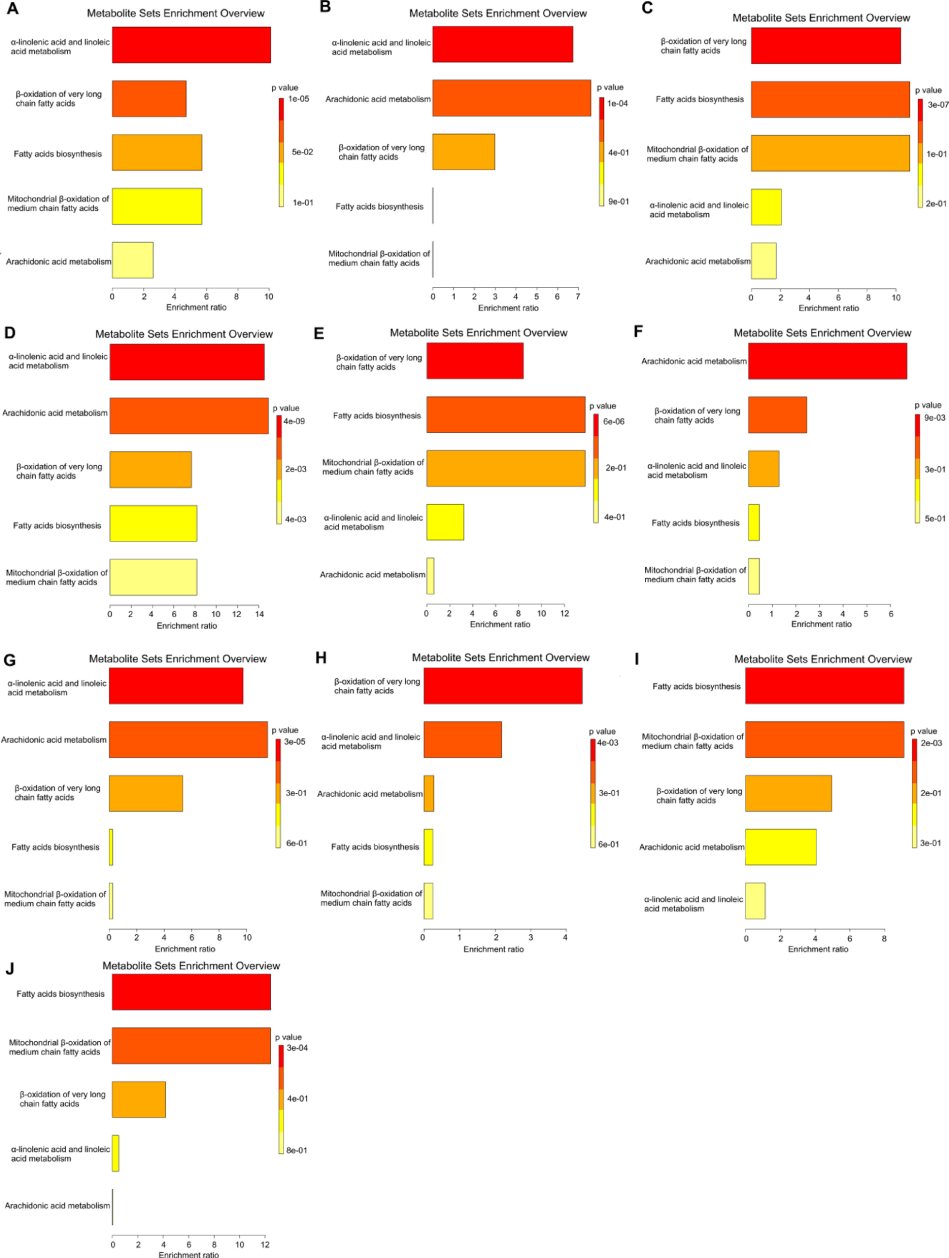
Summary plot of metabolite set enrichment analysis (MSEA) of highly correlated FAs: The horizontal bar graph summarizes the metabolic pathways that were significantly enriched by this group of functionally related FAs during the setting of I/R injury. MSEA indicating the 25 most disturbed metabolic pathways in (**A**) **HT** serum (comparison with control serum), (**B**) **HT** (comparison with FCA serum), (**C**) **FCA** (comparison with control serum), (**D**) **PTC** serum (comparison with HT serum), (**E**) **PTC** (comparison with FCA serum), (**F**) **PTC** (comparison with control serum), (**G**) **NG** serum (comparison with HT serum), (**H**) **NG** (comparison with FCA serum), (**I**) **NG** (comparison with control serum) and (**J**) **NG** serum (comparison with PTC serum).

To identify subtle but substantial changes among these correlated FAs with metabolic pathways, MSEA was performed for these functionally related metabolites together with their relative concentrations (Fig. 8). The second disease entity after HT in which serum FA profile had predictive power was FCA (0.796). Therefore, we wanted to examine in which pathways of FA metabolism in HT and FCA differ from control subjects, and whether they differ at all (Fig. 8). The key pathway in FA metabolism that was most significantly altered in HT comparing to controls was α-linolenic acid and linoleic acid metabolism (Fig. 8A), as well as when we compared HT vs. FCA (Fig. 8B). In turn in analysis of the FCA vs. HC β-oxidation of very long-chain fatty acids was changed to the greatest extent (Fig. 8C). β-oxidation of very long-chain fatty acids also changes in HT contra HC (Fig. 8A) but to a much lesser extent than when comparing FCA contra HC (Fig. 8C). Interestingly, change of arachidonic acid (ARA) metabolism in HT contra HC and FCA contra HC was very similar, hence this pathway is probably common to both thyroid diseases. In both diseases, there is a strong inflammatory state, which initiate the formation of pro-inflammatory mediators from ARA^18^. However, the highest differences in FAs metabolism were observed in PTC compared to HT, also in this case the largest number of FAs involved in the described metabolic pathways was determined (Fig. 8D). The smallest change in of arachidonic acid metabolism was observed when comparing PTC with FCA (Fig. 8E). The smallest changes in mitochondrial β-oxidation of medium chain fatty acids were observed when comparing PTC with HC (level 5 × 10⁻¹) (level 5 × 10⁻¹) (Fig. 8F), NG with HT (level 6 × 10⁻¹) (Fig. 8G), and NG with FCA (level 6 × 10⁻¹) (Fig. 8H). In turn, MSEA showed very similar patterns of changes in metabolic pathways during comparison NG vs. HC, and NG vs. PTC (Fig. 8I, J).

### Analysis of individual serum fatty acids depending on thyroid disease

Most FAs from the n3 family were significantly lower in serum of HT patients compared to controls (ALA, ETA, EPA, DHA), but also in PTC and FCA compared to HC. EPA was lower in NG patients compared to control and FCA (similar as DPA n3), as well as concentrations of DPA n3 and DHA were lower in NG compared to PTC. Based on these results, we can speculate about a disruption of the n3 synthesis pathway in HT patients (Fig. S8). In turn, the major serum FA of the n6 group were significantly lower in HT patients compared to PTC (DGLA, ARA, AdA, DPA n6). In addition, DGLA was also lower in FCA patients compared to PTC (Fig. S8). In all analyzed group of subjects, we didn’t observed difference between sum of total PUFA (*p* = 0.108). HT and NG are characterized by chronic inflammation, which can affect the level of SPMs in the patient’s blood^19^. However, analysis of whole group of n6 PUFA, shown no differences between analyzed groups of patients (Fig. S8). The next group of FAs considered were the VLCFAs. A significantly higher concentration of the whole group and individual FA (C22-C24) is observed in HT, FCA and PTC patients compared to controls (Fig. S9). Chronic inflammation causes symptoms not only in the organ affected by the disease but also affects the periphery. Although FAs are products of endogenous synthesis and are derived from the diet, our results clearly show that significant changes in the function of the altered thyroid gland due to the disease could affect the profile of FAs in the blood of patients. Total BCFAs, were significantly lower in serum of NG patients compared to FCA and HT (Fig. S9). In turn, acid iso C17, which was recorded at the highest concentration among whole BCFAs, was very close in each group of study. Anteiso C15 and anteiso C17 were significantly higher in HT compared to PTC and NG, however only anteiso C15 was higher in HT compared to control. The OCFA group consists mainly of C15 and C17, which originate from dairy products and ruminant meat^15^. FA with a larger number of C-atoms belonging to OC-VLCFA differed in NG from HT patients, but also from PTC (Fig. S10). Nevertheless, the HT patient group was characterized by a wide range of OCFA concentrations in their serum. In the group with total even-numbered FAs (ECFAs), no significant differences in concentration were detected among the studied groups of patients. Only the concentration of C12 in the serum of FCA and HT patients was significantly different from that in the control and PTC groups and stearic acid further distinguished the HT group from NG (Fig. S11). MUFAs as TAG components are important energy source. In our analysis there was not significant differences in MUFA between studied groups of patients, with a tendency to decrease in NG (Fig. S12).

## Discussion

Based on the results obtained, we found that (1) the total FA collections in the serum of patients with different thyroid diseases differed from each other, (2) the FA changes detected in the serum of HT are highly characteristic of this disease, allowing for the greatest differentiation of HT from other thyroid diseases, (3) metabolic alterations have been associated mainly with polyunsaturated FAs metabolism or with β-oxidation of VLCFA, (4) most alterations in individual FA are observed in the PUFA and VLCFA groups, (5) also, Hashimoto’s disease is the most diverse in terms of individual FAs, (6) although the FAs data from PTC did not show any pattern and had no predictive power in ROC analysis, this disease has the highest impact on FA metabolism compared to other diseases, with the greatest differences being observed when compared to HT in MSEA.

Thyroid diseases have emerged as a hallmark of the 21st century and, along with obesity, represent a significant public health challenge. Both external environmental factors and genetic predispositions, contribute to pathological conditions associated with hypothyroidism, hyperthyroidism and HT, which is often accompanied by a benign alteration or even a malignant thyroid tumor. Thyroid neoplasms can develop over extended periods without manifesting characteristic symptoms. A growing thyroid tumor is frequently indistinguishable from a benign nodule, and an enlarged thyroid gland often remains unnoticed by patients for a prolonged duration. Moreover, the current preoperative strategies for differentiating benign thyroid nodules from potentially malignant thyroid nodules remain unspecific. The only definitive method for early cancer detection is to perform a thyroid biopsy^20^. However, a less invasive approach, such as serum-based screening tests, may be a preferable alternative. Several studies increasingly highlight the significance of metabolomics as a potentially useful tool to differentiate malignant thyroid nodules from benign lesions^21–23^. Our research focused on FAs, a critical subset of metabolites that play a fundamental role in lipid metabolism. Oncogenic pathways influence lipid metabolism in tumors, including lipogenesis^24^. Regardless of the comparison groups, MSEA identifies a predominance of PUFA metabolism or β-oxidation of VLCFA and medium-chain FAs (Fig. 8). Thyroid hormones stimulate lipolysis of white adipose tissue, which is the main source of circulating free FAs^25^. Additionally, metabolic analyses of plasma from patients with PTC and multiNG compared to healthy individuals indicated an increase in FA-β oxidation, suggesting enhanced lipid metabolism in thyroid nodules^26^ and in PTC patients^14^. In line with these findings, Tain et al.^21^, who investigated metabolomics in plasma of PTC and multiNG patients, observed a reduction in lipid levels, which may indicate that lipolysis and FA oxidation are dominant bioenergetic pathways in thyroid disease^21^.

While HT can be easily differentiated based on thyroid antibodies, distinguishing thyroid tumors remains more challenging. To address this, we conducted a comprehensive series of ROC analyses, which demonstrated the potential for differentiating thyroid diseases with appropriate predictive power using a specific set of FAs. Additionally, MSEA identified diseases that can be distinguished from HT based solely on the overall FA profile in patient serum. Although HT may predispose individuals to thyroid malignancies, MSEA analysis revealed distinct FA metabolic pathways that predominate in each disease. Indeed, in HT, the specific FA set identified in other diseases may serve as a potential predictive biomarker set for disease classification. Interestingly, analysis of the individual FAs revealed no significant differences between the group of saturated long-chain FAs and the monounsaturated FAs, which are the fundamental components of every organism and primary dietary constituents. However, the alternations in FAs profile were observed in the groups of FAs associated with inflammation (PUFA, BCFA), alleviation anaemia, dyslipidemia, inflammation and fibrosis in vivo (OCFA)^27^, and in FAs, which are components of cell membranes affecting membrane fluidity, lipid droplet formation and lipid signalling pathways (VLCFAs)^28^. In the serum of healthy individuals, most FAs were present at lower concentrations than in the studied thyroid diseases (Fig. 1). Interestingly, whole representatives of VLCFAs were significantly lower in NG compared to HT. In HT, due to thyroid cell disintegration VLCFA can be released into the blood^29^. In NG, VLCFA can be taken up by the thyroid gland to form new cells in which colloid is stored, and thyroid hormones are overproduced^30^. In turn, the differences of OCFA in the blood of HT patients compared to NG could be due to the different diet to which HT patients are exposed^31,32^. Interestingly, in this disease it is worth limiting dairy products as they interfere with the absorption of drugs containing thyroid hormones, and casein also has a negative impact on the immune system^33^. Unfortunately, patients did not complete the FFQ questionnaire (one of the limitations of this paper), so this is just our speculation. ROC analysis identified predictive biomarker FAs, including n-3 PUFAs and VLCFA for HT (Fig. 3), VLCFAs and total anteiso BCFA for FCA (Fig. 5), and also 22:4 n6, C24:1, C19 and C14:1 for NG (Fig. 7). Our compilation of the broad FA profile in 4 major thyroid diseases is the first detailed study of its kind. Previously, the PUFA profile in serum of TC, thyroid adenoma (TA) and NG patients was analyzed^22^. Notably, our findings align with those of Li et al.^22^, who reported elevated adrenic acid in NG than in TC and TA. However, while the total sum of PUFAs to be highest in TA relative to TC and NG^22^, our study did not observe this significant difference.

Other lipidomics and metabolomics studies have reported comparable or divergent predictive performances for thyroid diseases. For instance, Li et al.^22^ and several Hashimoto’s thyroiditis metabolomics studies observed altered n-3 polyunsaturated fatty acids similar to our findings, with moderate discrimination (AUC ≈ 0.7–0.8). In contrast, plasma lipidomic panels for papillary thyroid carcinoma achieved higher accuracy (AUC > 0.9) when combining multiple lipid or metabolite markers^34^. These differences likely reflect disease type, analytical platforms, and whether single-analyte or multi-marker models were used.

A limitation of the present study is that all participants were female. Recruitment was intentionally limited to women, as the clinical population undergoing thyroid surgery at our center during the study period was predominantly female, and homogenous study groups were desired to enhance the reliability of between-group comparisons. From an epidemiological standpoint, thyroid disorders—including thyroid cancer, Hashimoto’s thyroiditis, hypothyroidism, and goiter—are substantially more prevalent in women than in men, which further supports the female predominance observed in our cohort^7,11^. Nonetheless, future studies should aim to include male participants to determine whether the alterations in FA profiles reported here exhibit sex-dependent patterns. Furthermore, concomitant use of certain medications (e.g., levothyroxine, lipid-lowering, or antidiabetic agents) as well as dietary habits may modulate circulating lipid and FA profiles^7^. While the principal objective of this study was to delineate differences primarily related to thyroid pathology, these factors should be acknowledged as potential confounders and merit consideration in future research designs.

The novelty in this work is the FA analysis not only of nodules patients and confirmation of the changes in FA metabolism compared to healthy subjects, but above all the inclusion of HT in the analysis of test results, which often accompanies PTC. Having all variants of thyroid lesions available, we are more aware of the changes in FA composition in serum including their deficiencies or excesses. These findings underscore the complexity of FA metabolism in thyroid diseases and highlight the potential FA profiling as a diagnostic and predictive tool.

## Conclusion

We examined the patients’ serum, which FA composition is dependent on the metabolic processes in the body as well as the composition of ingested food. Serum FA patterns are strongly influenced by thyroid dysfunction caused by thyroid disease. Our study provides further insight into the pathophysiology of thyroid dysfunction caused by the four major thyroid diseases. However, longitudinal studies with a larger population are required to confirm these findings and further research is urgently needed to gain better insights into the underlying disease mechanisms.

## Supplementary Information

Below is the link to the electronic supplementary material.

Supplementary Material 1

## Data Availability

The datasets used and/or analysed during the current study are available from the corresponding author on reasonable request.
