# Supplementary material for: Different fatty acid patterns in serum in patients with various thyroid diseases
Source: Sci Rep. 2026 Jan 5;16:3812. doi: 10.1038/s41598-025-33974-9 (PMC12852874; doi:10.1038/s41598-025-33974-9)
Supplement: Supplementary file 1 — Supplementary Material 1 [file 41598_2025_33974_MOESM1_ESM.docx]

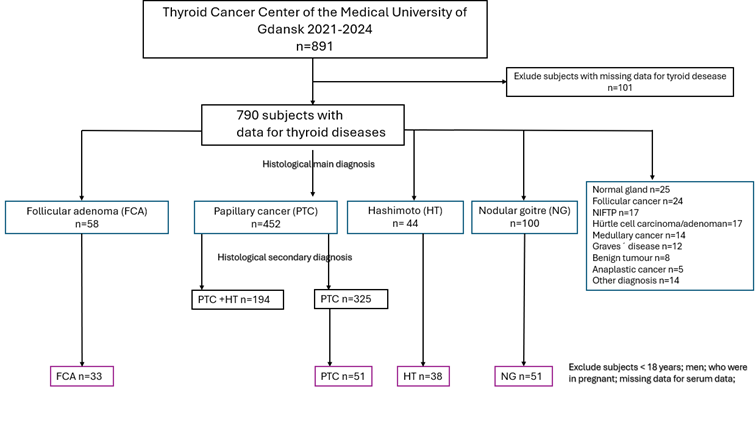


**Figure S1:** Flowchart of study inclusion.

**Table 1**: Anthropometric, clinical and laboratory parameters of examined patients.

|  |  |  |  |  |  | **p-value** | | | | | | | | | |
| --- | --- | --- | --- | --- | --- | --- | --- | --- | --- | --- | --- | --- | --- | --- | --- |
|  | Control | PTC | FCA | NG | HT | HC vs PTC | HC vs FCA | HC vs NG | HC vs HT | PTC vs FCA | PTC vs HT | PTC vs NG | FCA vs NG | FCA vs HT | NG vs HT |
| Age (years) | 43.8 ± 12.8 | 42.4 ± 11.0 | 49.5 ± 12.9 | 49.0 ± 11.7 | 42.2 ± 9.80 | ns | ns | ns | ns | ns | ns | ns | ns | ns | ns |
| BMI (kg/m^2^) | 25.4 ± 7.2 | 28.8 ± 7.32 | 26.3 ± 5.94 | 27.5 ± 5.89 | 25.8 ± 4.85 | ns | ns | ns | ns | ns | ns | ns | ns | ns | ns |
| TG (mg/dL) | 104 ± 61.4 | 112 ± 58.6 | 101 ± 52.8 | 102 ± 48.1 | 101 ± 52.1 | ns | ns | ns | ns | ns | ns | ns | ns | ns | ns |
| HDL (mg/dL) | 57.9 ± 10.9 | 55.2 ± 14.6 | 56.7 ± 10.6 | 54.5 ± 13.9 | 51.9 ± 15.5 | ns | ns | ns | ns | ns | ns | ns | ns | ns | ns |
| LDL (mg/dL) | 124 ± 38.5 | 133 ± 40.2 | 116 ± 37.7 | 119 ± 32.1 | 114 ± 33.9 | ns | ns | ns | ns | ns | ns | ns | ns | ns | ns |
| TC (mg/dL) | 203 ± 45.8 | 203 ± 32.0 | 193 ± 38.6 | 194 ± 38.7 | 209 ± 41.3 | ns | ns | ns | ns | ns | ns | ns | ns | ns | ns |
| CRP (mg/L) | 2.47 ± 3.59 | 1.80 ± 1.32 | 1.24 ± 1.59 | 1.75 ± 1.75 | 4.10 ± 3.31 | ns | ns | ns | 0.004 | 0.035 | ns | ns | ns | < 0.001 | ns |
| Glucose (mg/dL) | ND | 93.8 ± 17.5 | 97.2 ± 17.8 | 104 ± 43.1 | 89.6 ± 26.0 | ns | ns | ns | ns | ns | ns | ns | ns | ns | ns |
| HBA1C (%) | 5.5 ± 0.23 | 5.4 ± 0.38 | 5.4 ± 0.36 | 5.8 ± 1.29 | 5.5 ± 0.34 | ns | ns | ns | ns | ns | ns | ns | ns | ns | ns |
| Insulin (uU/mL) | 8.1 ± 5.99 | 15.8 ± 20.0 | 13.1 ± 16.9 | 13.1 ± 10.6 | 10.4 ± 10.1 | ns | ns | ns | ns | ns | ns | ns | ns | ns | ns |
| Albumin (g/L) | 40.8 ± 2.96 | 42.6 ± 4.21 | 42.1 ± 4.53 | 40.1 ± 8.00 | 41.4 ± 3.28 | ns | ns | ns | ns | ns | ns | ns | ns | ns | ns |
| Creatinine (mg/dL) | 0.72 ± 0.11 | 0.70 ± 0.09 | 0.72 ± 0.12 | 0.70 ± 0.16 | 0.73 ± 0.11 | ns | ns | ns | ns | ns | ns | ns | ns | ns | ns |
| 1,25-(OH)_2_D (pg/mL) | 44.9 ± 16.5 | 53.2 ± 14.8 | 53.4 ± 21.7 | 55.6 ± 19.0 | 52.3 ± 16.5 | ns | ns | ns | ns | ns | ns | ns | ns | ns | ns |
| TSH (uU/mL) | 1.67 ± 0.87 | 1.08 ± 0.89 | 1.11 ± 1.04 | 0.90 ± 0.57 | 2.54 ± 1.87 | 0.031 | 0.046 | ns | ns | ns | < 0.001 | ns | ns | < 0.001 | 0.006 |
| fT3 (pmol/L) | 4.30 ± 0.60 | 4.33 ± 0.68 | 4.29 ± 0.69 | 4.52 ± 0.59 | 3.99 ± 0.57 | ns | ns | ns | ns | ns | ns | ns | ns | ns | ns |
| fT4 (pmol/L) | 12.2 ± 1.23 | 11.9 ± 2.02 | 12.1 ± 1.65 | 11.8 ± 1.77 | 12.6 ± 3.78 | ns | ns | 0.023 | ns | ns | ns | ns | ns | ns | 0.012 |
| TPOAb (IU/L) | <3.00 | 9.7 ± 41.7 | 188 ± 713 | 72.5 ± 176 | 218 ± 287 | ns | ns | ns | < 0.001 | ns | 0.002 | ns | ns | ns | ns |
| TgAb (IU/L)  *Drugs*  Levothyroxine  Thiamazolum  Rosuvastatin + Ezetimibe  Rosuvastatin  Atorvastatin + Ezetimibe  Atorvastatin  Simvastatin  Fenofibratum  *Allergies*  *Comorbidities*  Obesity  Dyslipidemia  Diabetes  Insulin resistance  Hypertension | <3.00  0 (0.0%)  0 (0.0%)  0 (0.0%)  0 (0.0%)  0 (0.0%)  0 (0.0%)  0 (0.0%)  0 (0.0%)  1 (3.2%)  4 (12.9%)  4 (12.9%)  0 (0.0%)  0 (0.0%)  1 (3.2%) | 4.7 ± 11.1  20 (40.0%)  0 (0.0%)  0 (0.0%)  3 (6.0%)  0 (0.0%)  0 (0.0%)  0 (0.0%)  0 (0.0%)  1 (2.0%)  15 (30.0%)  3 (6.0%)  1 (2.0%)  2 (4.0%)  6 (12%) | 46.7 ± 144  8 (25.0%)  0 (0.0%)  1 (3.1%)  1 (3.1%)  0 (0.0%)  1 (3.1%)  1 (3.1%)  1 (3.1%)  5 (15.6%)  8 (25.0%)  4 (12.5%)  1 (3.1%)  2 (6.3%)  8 (25.0%) | 105 ± 289  5 (9.8%)  14 (27.5%)  0 (0.0%)  4 (7.8%)  1 (2.0%)  1 (2.0%)  0 (0.0%)  0 (0.0%)  3 (5.9%)  13 (25.5%)  6 (11.8%)  3 (5.9%)  1 (2.0%)  9 (17.6%) | 79.8 ± 129  6 (15.8%)  0 (0.0%)  0 (0.0%)  0 (0.0%)  0 (0.0%)  0 (0.0%)  0 (0.0%)  0 (0.0%)  3 (7.9%)  6 (15.8%)  1 (2.6%)  1 (2.6%)  1 (2.6%)  1 (2.6%) | ns | ns | ns | < 0.001 | ns | < 0.001 | ns | ns | ns | ns |

1,25-(OH)_2_D – Calcitriol, TgAbs – Thyroglobulin antibodies, TPOAbs – Thyroid peroxidase antibodies, BMI – Body mass index, CRP – C-reactive protein, FCA – Follicular-cell adenoma, fT3 – Free triiodothyronine, fT4 – Free thyroxine, HbA1C – Hemoglobin A1c, HC – Healthy control, HDL – High-density lipoprotein, HT – Hashimoto’s thyroiditis, LDL – Low-density lipoprotein, ND - no data, NG – Nodular goiter, ns – not significant, TC – Total cholesterol , TG – Triglycerides, TSH – Thyroid stimulating hormone, PTC – Papillary thyroid cancer

**
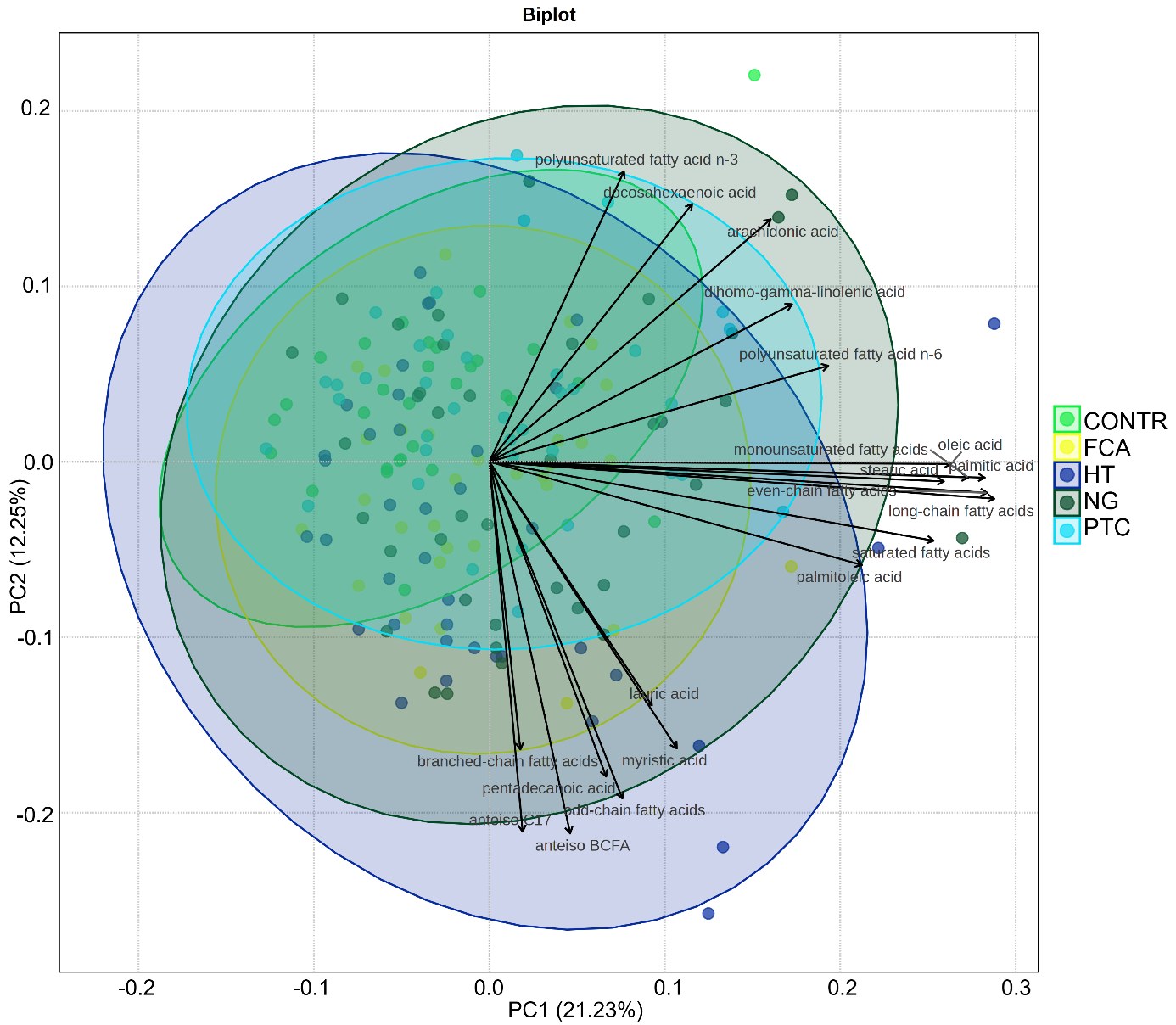
**

**Figure S2**: The results of principal component analysis (PCA) of individuals based on the whole fatty acid profile: score plot of cases for first two PCs.

**Table 2:** Classical ROC curve analysis for individual biomarkers when the target group is HT.

| **Name** | **AUC** | **T-test** | **Log2fc** |
| --- | --- | --- | --- |
| α-linolenic acid | 0.77255 | 7.8395E-6 | -0.69084 |
| heneicosanoic acid | 0.77135 | 3.6585E-9 | 0.85134 |
| eicosapentaenoic acid | 0.77061 | 1.9145E-5 | -0.59073 |
| docosahexaenoic acid | 0.76517 | 2.5159E-7 | -0.56396 |
| polyunsaturated fatty acids n-3 | 0.76388 | 1.2306E-6 | -0.49589 |
| lignoceric acid | 0.76241 | 1.1111E-6 | 0.46395 |
| very long-chain fatty acids | 0.75042 | 6.0214E-5 | 0.39501 |
| nonadecanoic acid | 0.73409 | 1.5242E-6 | 0.62901 |
| docosapentaenoic acid n-3 | 0.72699 | 5.4234E-4 | -0.27623 |
| arachidonic acid | 0.7257 | 4.0567E-5 | -0.23585 |
| behenic acid | 0.70079 | 0.0079019 | 0.34129 |
| arachidic acid | 0.6877 | 1.1874E-4 | 0.49887 |
| adrenic acid | 0.68751 | 4.9501E-4 | -0.25003 |
| docosapentaenoic acid n-6 | 0.68696 | 8.1626E-4 | -0.32958 |
| odd chain very long fatty acids | 0.67285 | 0.0015311 | 0.37704 |
| dihomo-gamma-linolenic acid | 0.66159 | 0.0024426 | -0.15998 |
| nervonic acid | 0.65975 | 8.2547E-4 | 0.44218 |
| eicosatetraenoic acid | 0.6579 | 0.0061662 | -0.20123 |
| sum of polyunsaturated fatty acids | 0.63761 | 0.014599 | -0.079272 |
| odd-chain fatty acids | 0.62516 | 0.0024177 | 0.4186 |
| cerotic acid | 0.61834 | 0.0036097 | 0.5734 |
| anteiso C17 | 0.61538 | 0.020378 | 0.45379 |
| polyunsaturated fatty acid n6 | 0.6104 | 0.012086 | -0.049062 |
| tricosanoic acid | 0.59721 | 0.23354 | 0.24342 |
| nonadecenoic acid | 0.59196 | 0.028975 | 0.44897 |
| anteiso branched-chain fatty acids | 0.59094 | 0.14244 | 0.30964 |
| tridecanoic acid | 0.57849 | 0.031037 | 0.53711 |
| pentadecanoic acid | 0.57757 | 0.021326 | 0.4442 |
| lauric acid | 0.56761 | 0.013705 | 0.67933 |
| iso C14 | 0.56336 | 0.51465 | 0.30854 |
| heptadecanoic acid | 0.56207 | 0.087055 | 0.27223 |
| myristoleic acid | 0.54916 | 0.10636 | -0.065429 |
| palmitoleic acid | 0.54916 | 0.027929 | 0.35762 |
| iso C17 | 0.53403 | 0.52781 | 0.1051 |
| anteiso C15 | 0.53302 | 0.43164 | 0.31126 |
| iso C15 | 0.5321 | 0.80436 | 0.097459 |
| iso branched-chain fatty acids | 0.53099 | 0.44599 | 0.1037 |
| myristic acid | 0.52693 | 0.58829 | 0.2399 |
| oleic acid | 0.52167 | 0.58101 | 0.098059 |
| linoleic acid | 0.5178 | 0.95194 | 0.13891 |
| eicosenoic acid | 0.51771 | 0.54145 | 0.17588 |
| palmitic acid | 0.51577 | 0.77188 | 0.14761 |
| sum of saturated fatty acids | 0.51503 | 0.20778 | 0.22439 |
| eicosadienoic acid | 0.51208 | 0.70754 | 0.092634 |
| sum of branched-chain fatty acids | 0.51024 | 0.63291 | 0.087985 |
| sum of monounsaturated fatty acids | 0.50895 | 0.89985 | 0.12613 |
| long-chain fatty acids | 0.50636 | 0.55325 | 0.16906 |
| stearic acid | 0.50618 | 0.18766 | 0.21511 |
| iso C16 | 0.50341 | 0.92215 | 0.17732 |
| even-chain fatty acids | 0.50323 | 0.43948 | 0.18114 |

The features displayed in the table below are ranked based on the area under ROC curve (AUROC), T-statistics and Log2 fold change (FC). The 95% confidence interval is calculated using 500 boot strappings. Additional parameters: threshold 0.2 and optimal cutoff using closest to top-left corner.


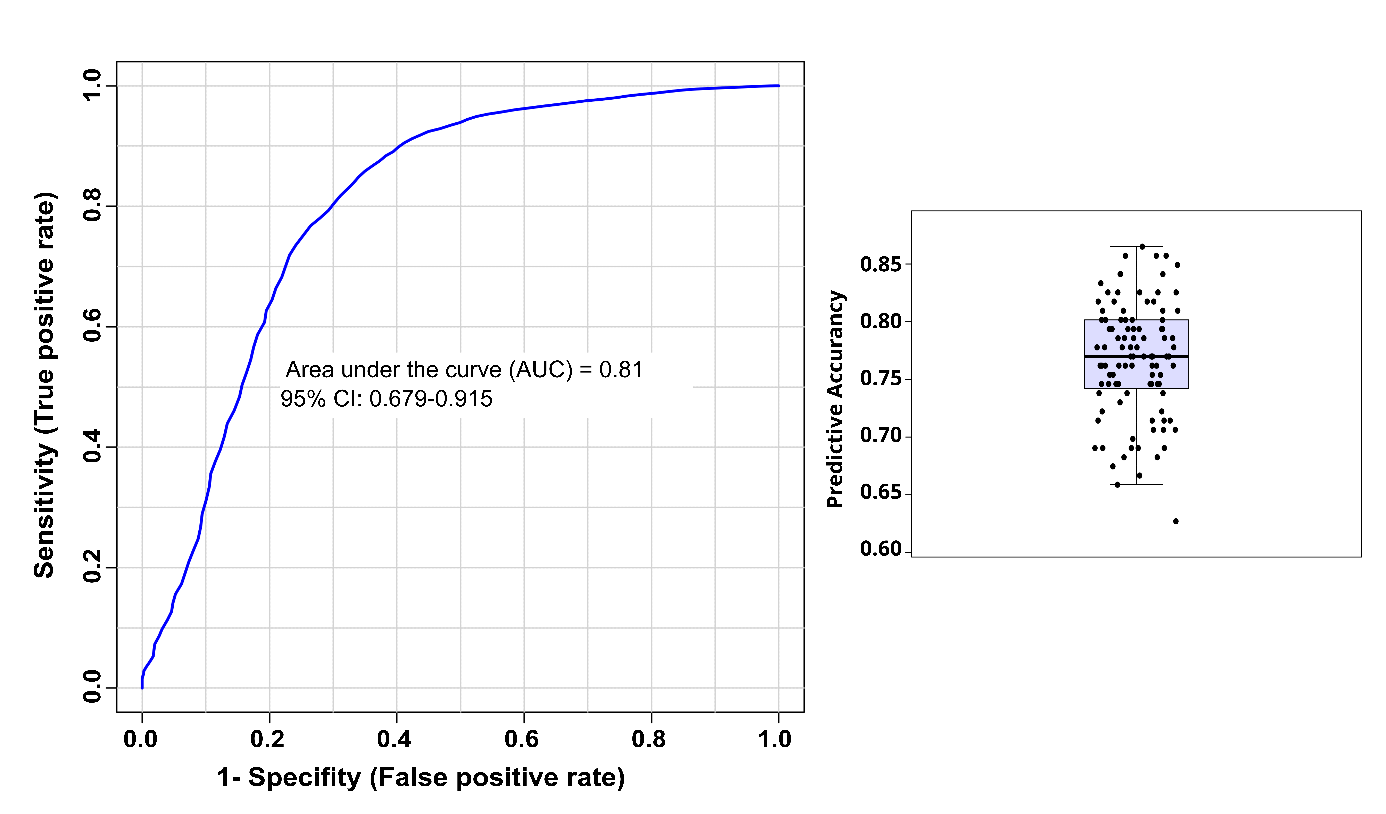


**Figure S3:** ROC curve by selecting FAs to predict HT vs other analyzed groups.


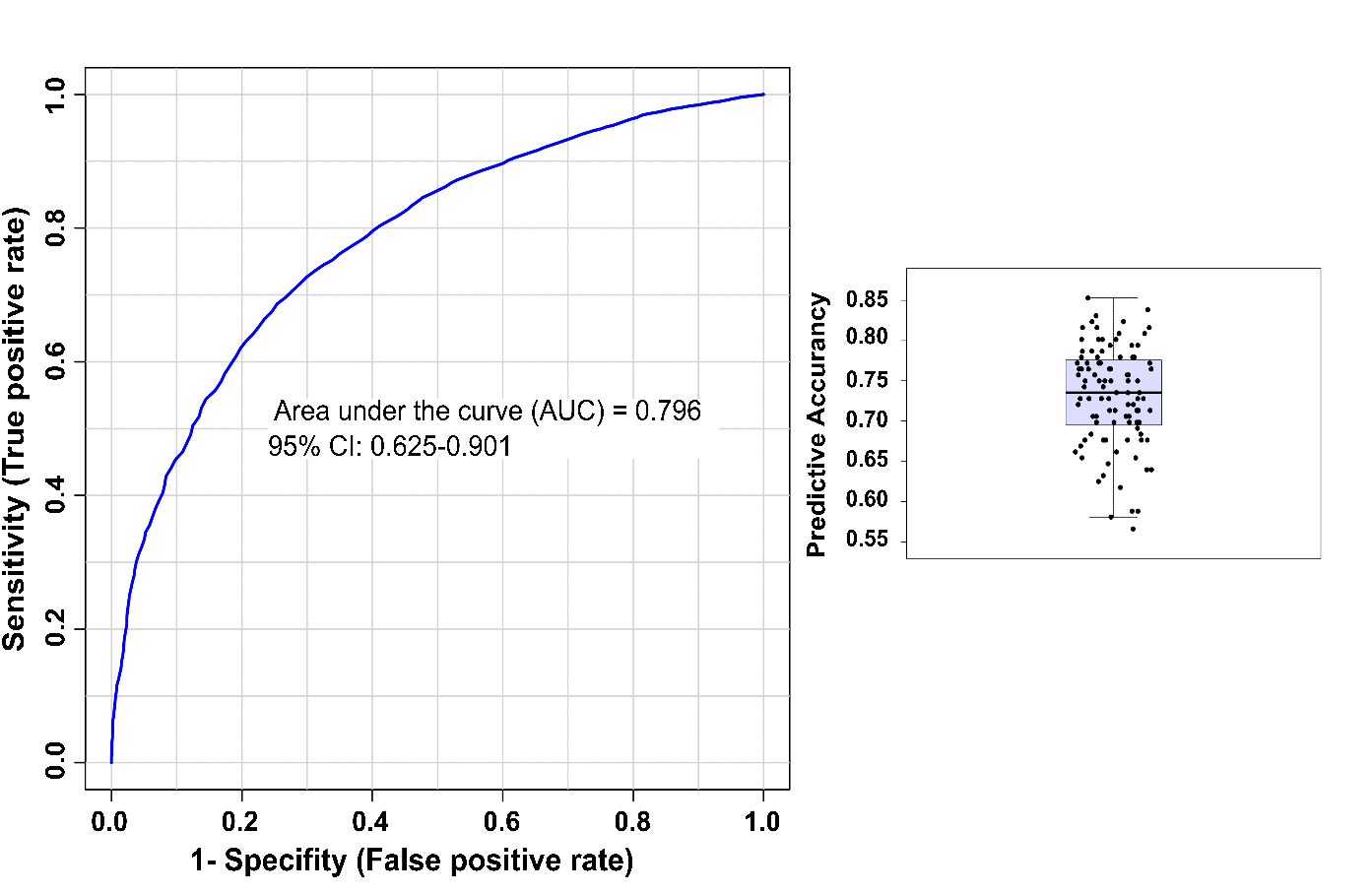


**Figure S4:** ROC curve by selecting FAs to predict FCA vs other analyzed groups.

**Table 3:** Classical ROC curve analysis for individual biomarkers when the target group is FCA.

| **Name** | **AUC** | **T-test** | **Log2fc** |
| --- | --- | --- | --- |
| nervonic acid | 0.69403 | 0.0014407 | -0.34501 |
| cerotic acid | 0.68493 | 1.6873E-4 | 0.62741 |
| lignoceric acid | 0.67947 | 0.0087358 | -0.20955 |
| anteiso branched-chain fatty acids | 0.67241 | 0.0037987 | 0.35235 |
| branched-chain fatty acids | 0.6555 | 0.0075732 | 0.28012 |
| nonadecanoic acid | 0.65218 | 0.008697 | 0.34988 |
| iso C14 | 0.64983 | 0.010775 | -0.43006 |
| polyunsaturated fatty acid n-3 | 0.64298 | 0.012965 | 0.31024 |
| nonadecenoic acid | 0.64148 | 0.0053103 | 0.40058 |
| anteiso C15 | 0.63806 | 0.013526 | 0.36592 |
| dihomo-gamma-linolenic acid | 0.63602 | 0.010376 | -0.2075 |
| eicosenoic acid | 0.6357 | 0.035821 | 0.26089 |
| lauric acid | 0.63099 | 0.16446 | 0.36248 |
| eicosatetraenoic acid | 0.63035 | 0.010711 | -0.25405 |
| tridecanoic acid | 0.63024 | 0.031115 | 0.38814 |
| docosapentaenoic acid n-3 | 0.62232 | 0.093285 | 0.20718 |
| anteiso C17 | 0.61965 | 0.046366 | 0.29512 |
| odd chain very long chain fatty acids | 0.61622 | 0.079024 | -0.13862 |
| eicosapentaenoic acid | 0.61066 | 0.051418 | 0.30002 |
| behenic acid | 0.60006 | 0.15639 | -0.11684 |
| very long-chain fatty acids | 0.5975 | 0.18679 | -0.08769 |
| eicosadienoic acid | 0.59407 | 0.07495 | -0.16408 |
| iso C15 | 0.58958 | 0.16783 | 0.20426 |
| iso C17 | 0.58818 | 0.22416 | 0.1656 |
| docosahexaenoic acid | 0.58765 | 0.18497 | 0.19543 |
| arachidic acid | 0.58433 | 0.17595 | -0.14539 |
| Tricosanoic acid | 0.5838 | 0.30673 | -0.065814 |
| adrenic acid | 0.57277 | 0.26051 | -0.048891 |
| palmitoleic acid | 0.55255 | 0.16227 | -0.15202 |
| polyunsaturated fatty acids | 0.5488 | 0.25908 | 0.10744 |
| iso branched-chain fatty acids | 0.54677 | 0.7151 | 0.07636 |
| arachidonic acid | 0.54259 | 0.84554 | 0.047471 |
| stearic acid | 0.54259 | 0.2976 | -0.061566 |
| pentadecanoic acid | 0.54227 | 0.92898 | -3.9149E-4 |
| linoleic acid | 0.54024 | 0.49142 | -0.052358 |
| myristic acid | 0.54024 | 0.70501 | 0.056599 |
| monounsaturated fatty acids | 0.53981 | 0.39283 | -0.05156 |
| oleic acid | 0.53671 | 0.45609 | -0.042469 |
| heneicosanoic acid | 0.5305 | 0.29268 | -0.16047 |
| iso C16 | 0.52922 | 0.26719 | -0.094726 |
| heptadecanoic acid | 0.52836 | 0.96472 | 0.021512 |
| alpha-linolenic acid | 0.52322 | 0.33395 | -0.12724 |
| long-chain fatty acids | 0.51969 | 0.51664 | -0.030491 |
| even-chain fatty acids | 0.51969 | 0.4423 | -0.042399 |
| docosapentaenoic acid n-6 | 0.5153 | 0.66074 | -0.031413 |
| odd-chain fatty acids | 0.51113 | 0.35506 | -0.085272 |
| palmitic acid | 0.50985 | 0.56744 | -0.027437 |
| polyunsaturated fatty acid n-6 | 0.50706 | 0.86866 | 0.0030736 |
| saturated fatty acids | 0.50364 | 0.69135 | -0.0094791 |
| Myristoleic acid | 0.50139 | 0.50121 | -0.069246 |

The features displayed in the table below are ranked based on the area under ROC curve (AUROC), T-statistics and Log2 fold change (FC). The 95% confidence interval is calculated using 500 boot strappings. Additional parameters: threshold 0.2 and optimal cutoff using closest to top-left corner.


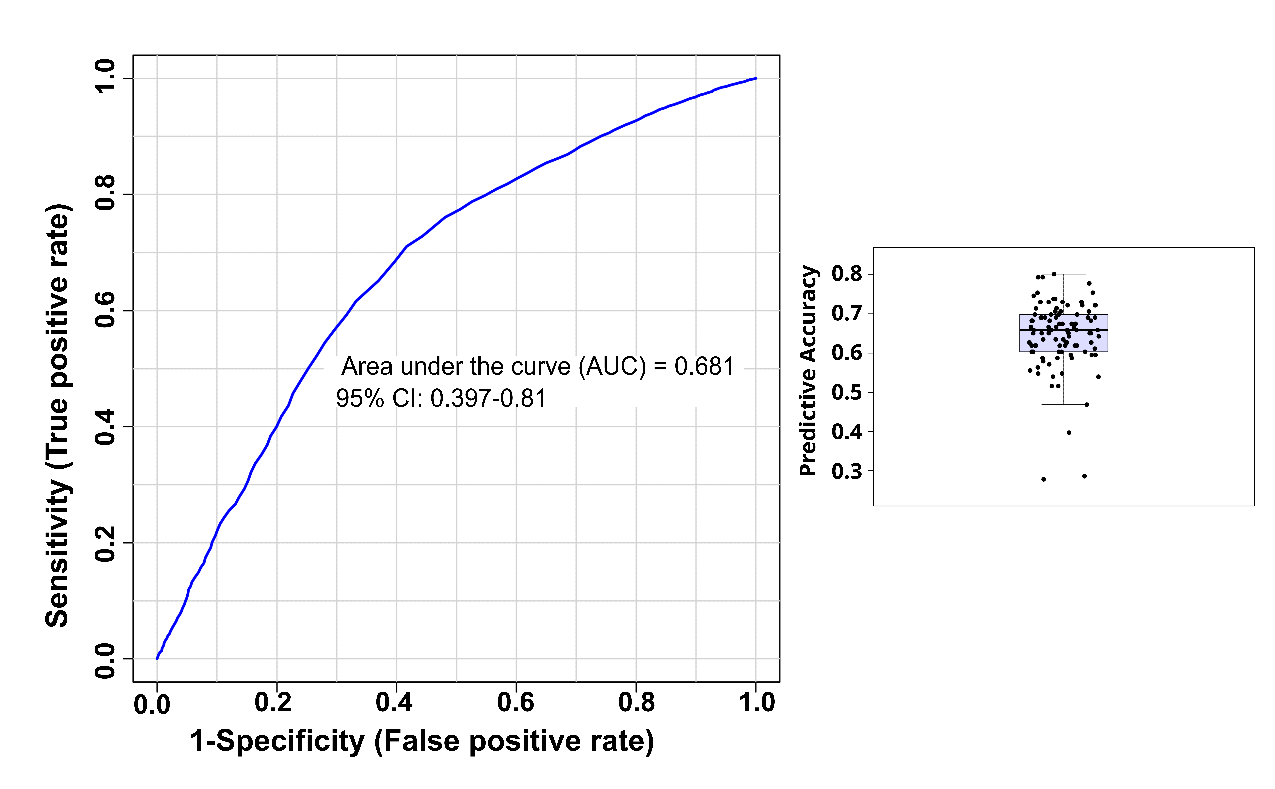


**Figure S5:** ROC curve by selecting FAs to predict NG vs other analyzed groups.

**Table 4:** Classical ROC curve analysis for individual biomarkers when the target group is NG.

| **Name** | **AUC** | **T-test** | **Log2fc** |
| --- | --- | --- | --- |
| adrenic acid | 0.65717 | 0.006046 | -0.061168 |
| nervonic acid | 0.65615 | 0.010138 | -0.55331 |
| nonadecanoic acid | 0.65569 | 0.0048827 | -0.66475 |
| myristoleic acid | 0.65458 | 3.4924E-5 | 0.31972 |
| arachidonic acid | 0.63365 | 0.0018596 | -0.022253 |
| iso C14 | 0.63254 | 0.0073987 | 0.029846 |
| lauric acid | 0.62747 | 0.08848 | 0.082887 |
| eicosatetraenoic acid | 0.62322 | 0.011187 | -0.074784 |
| dihomo-gamma-linolenic acid | 0.61658 | 0.0043995 | -0.052549 |
| pentadecanoic acid | 0.61631 | 0.1741 | -0.14496 |
| saturated fatty acids | 0.61548 | 0.045032 | -0.098993 |
| tricosanoic acid | 0.61538 | 0.035275 | -0.48322 |
| nonadecenoic acid | 0.60884 | 0.013465 | -0.70402 |
| long-chain fatty acids | 0.60782 | 0.056083 | -0.11983 |
| tridecanoic acid | 0.60561 | 0.21539 | -0.14918 |
| even-chain fatty acids | 0.60395 | 0.085256 | -0.12727 |
| eicosenoic acid | 0.60284 | 0.073427 | -0.50235 |
| palmitic acid | 0.60137 | 0.05565 | -0.10903 |
| polyunsaturated fatty acid n6 | 0.60044 | 0.0053166 | -0.085426 |
| monounsaturated fatty acids | 0.5962 | 0.032651 | -0.098265 |
| oleic acid | 0.59454 | 0.018963 | -0.083773 |
| stearic acid | 0.58753 | 0.15905 | -0.15935 |
| iso C15 | 0.58486 | 0.08885 | -0.048455 |
| anteiso C17 | 0.58209 | 0.3769 | -0.35847 |
| alpha-linolenic acid | 0.57729 | 0.2031 | -0.081792 |
| odd chain very long chain fatty acids | 0.57572 | 0.12542 | -0.42453 |
| very long-chain fatty acids | 0.57508 | 0.043678 | -0.4523 |
| odd-chain fatty acids | 0.57397 | 0.48165 | -0.20965 |
| anteiso C15 | 0.57388 | 0.25998 | -0.13373 |
| myristic acid | 0.57305 | 0.24571 | -0.10622 |
| behenic acid | 0.57047 | 0.28926 | -0.37786 |
| palmitoleic acid | 0.56567 | 0.79374 | -0.22658 |
| linoleic acid | 0.56023 | 0.056996 | -0.12953 |
| polyunsaturated fatty acid | 0.55423 | 0.22289 | -0.19069 |
| cerotic acid | 0.55396 | 0.031241 | -0.68076 |
| iso branched-chain fatty acids | 0.54935 | 0.24848 | -0.22297 |
| docosapentaenoic acid n-6 | 0.5439 | 0.64476 | -0.25432 |
| docosahexaenoic acid | 0.54372 | 0.11398 | -0.097193 |
| iso C16 | 0.53616 | 0.87289 | -0.34466 |
| anteiso branched-chain fatty acids | 0.53542 | 0.81033 | -0.2264 |
| iso C17 | 0.53505 | 0.63067 | -0.34909 |
| heptadecanoic acid | 0.53062 | 0.95873 | -0.25851 |
| branched-chain fatty acids | 0.52822 | 0.75558 | -0.32887 |
| docosapentaenoic acid n-3 | 0.52527 | 0.24886 | -0.41265 |
| eicosapentaenoic acid | 0.51752 | 0.778 | -0.27817 |
| heneicosanoic acid | 0.51716 | 0.76798 | -0.29075 |
| eicosadienoic acid | 0.51697 | 0.96244 | -0.31183 |
| polyunsaturated fatty acid n-3 | 0.5155 | 0.84535 | -0.33538 |
| lignoceric acid | 0.5059 | 0.46092 | -0.35279 |
| arachidic acid | 0.50424 | 0.49606 | -0.36344 |

The features displayed in the table below are ranked based on the area under ROC curve (AUROC), T-statistics and Log2 fold change (FC). The 95% confidence interval is calculated using 500 boot strappings. Additional parameters: threshold 0.2 and optimal cutoff using closest to top-left corner.


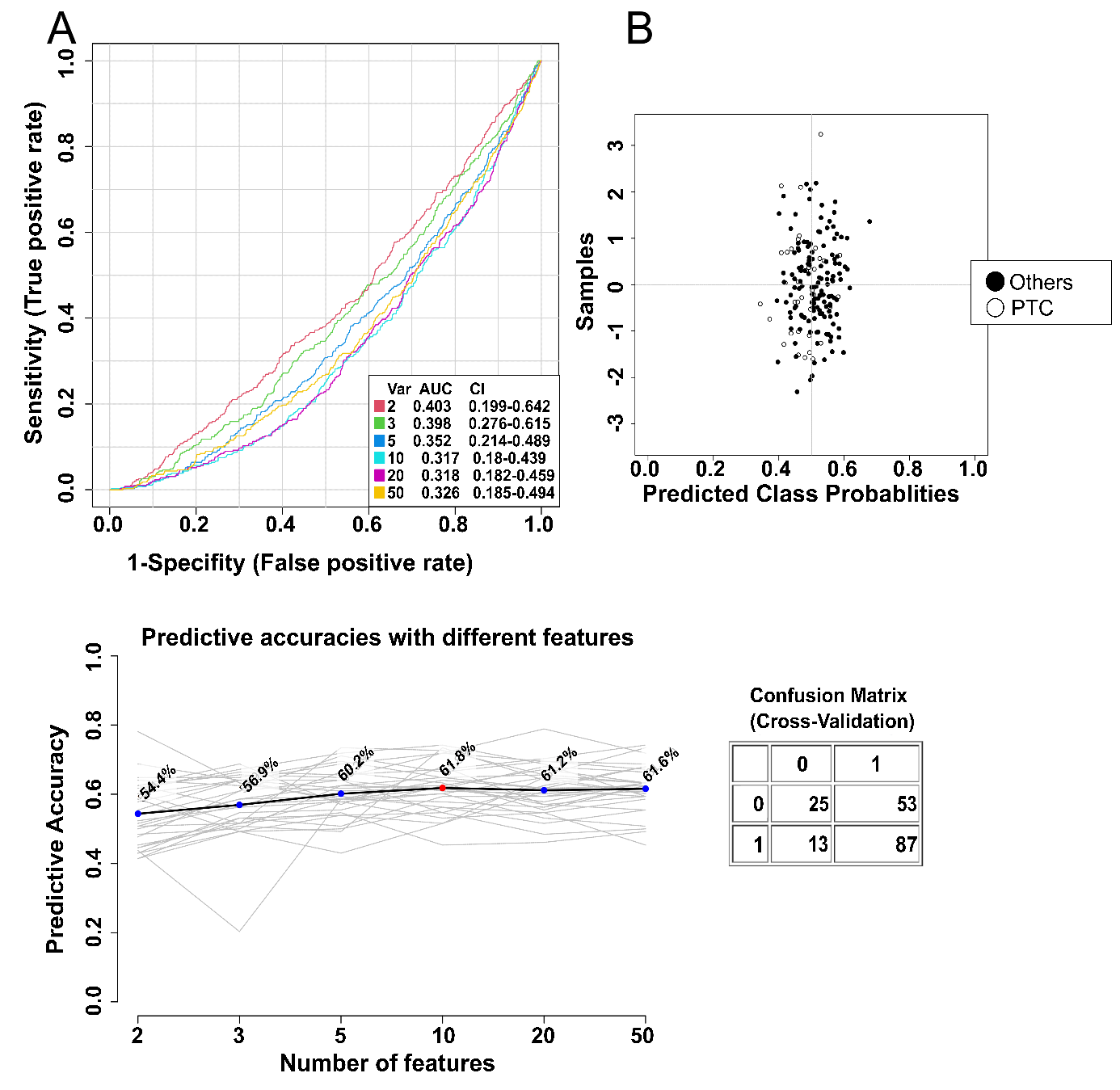


**Figure S6:** ROC curve of FA indicators from serum predicting the occurrence of papillary thyroid cancer (PTC) disease.


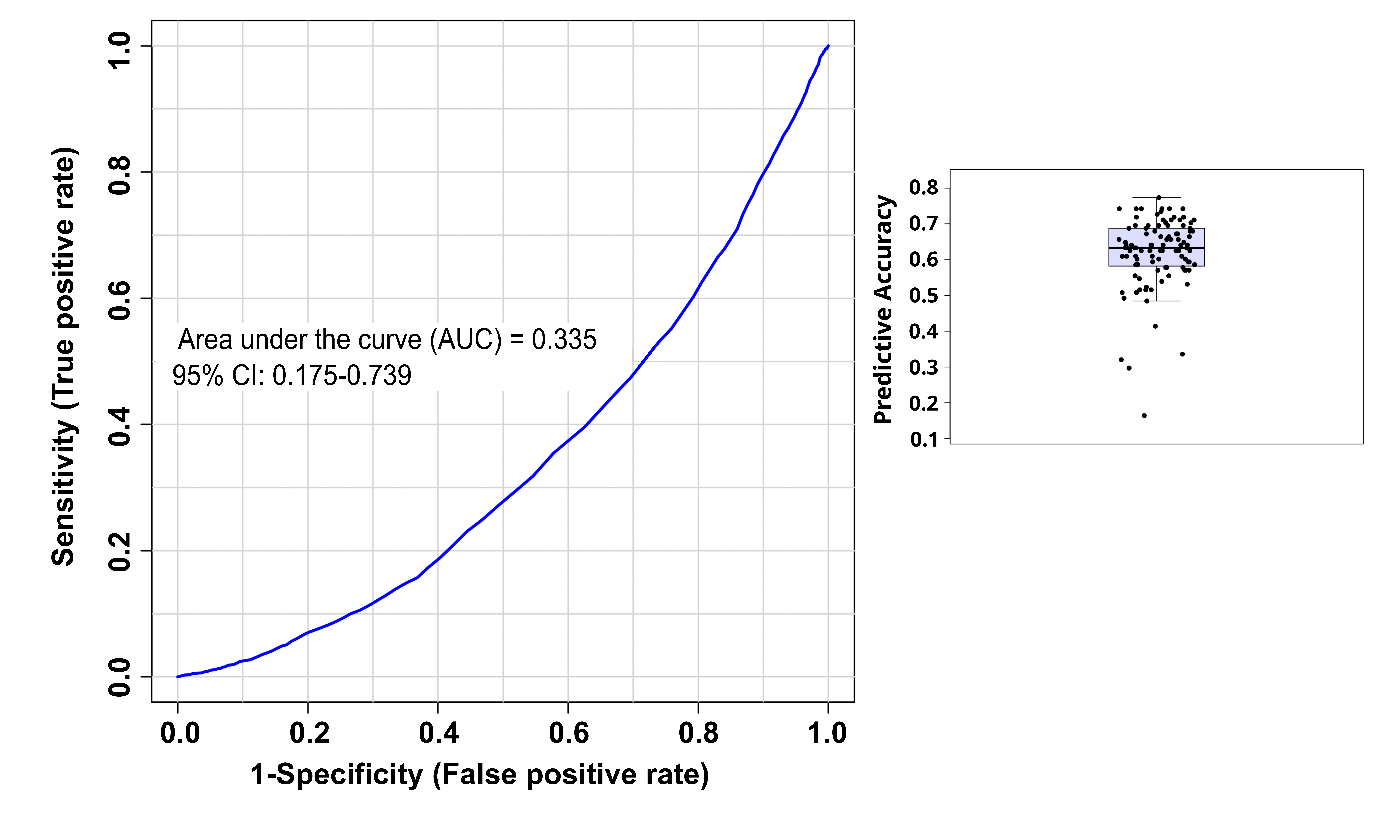


**Figure S7:** ROC curve by selecting FAs to predict PTC vs other analyzed groups.


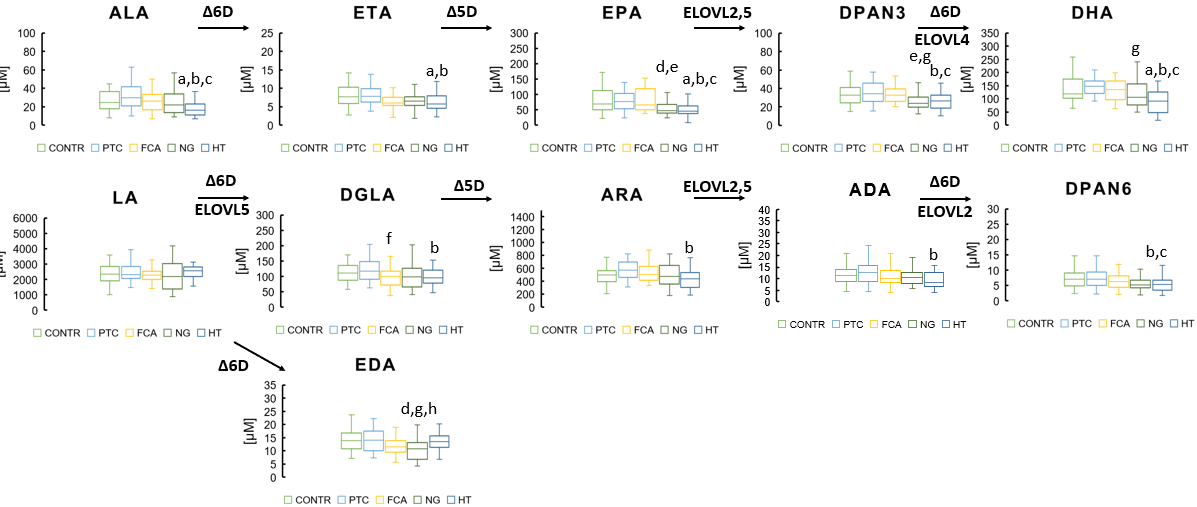


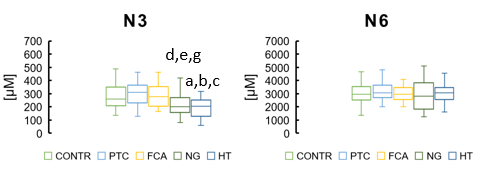


**Figure S8:** Polyunsaturated fatty acids concentrations in study subjects’ serum. N3 – n3 PUFA, N6 - n6 PUFA, ALA - α-linolenic acid, ETA – eicosatetraenoic acid, EPA – eicosapentaenoic acid, DPAn3 – docosapentaenoic acid n3, DHA – docosahexaenoic acid, LA – linoleic acid, DGLA - dihomo-γ-linolenic acid, ARA – arachidonic acid, AdA – adrenic acid, DPAn6 – docosapentaenoic acid n6, EDA – eicosadienoic acid, Δ5D – delta-5 desaturase, Δ6D- delta-6 desaturase, ELOVL2,4,5 - fatty acid elongase 2,4,5. **a** – HT vs. control; **b** - HT vs. PTC; **c** – HT vs. FCA; **d** – NG vs. control; **e** – NG vs. FCA; **f** – PTC vs. FCA; **g** – PTC vs. NG; **h** – HT vs. NG; **i** – FCA vs. control.


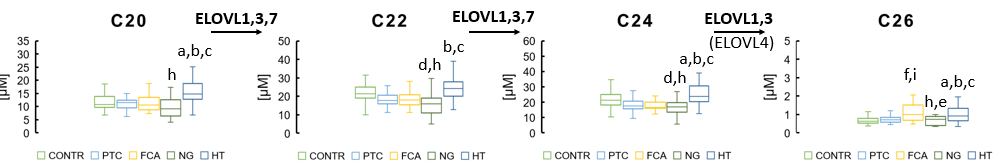


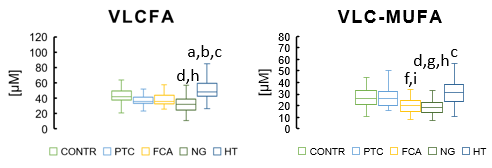


**Figure S9:** Fatty acid concentrations in study subjects’ serum. VLCFA – very long-chain FAs, VLC-MUFA – very long-chain monounsaturated FAs, ELOVL1,3,4,7 - fatty acid elongase 1,3,4,7. **a** – HT vs. control; **b** - HT vs. PTC; **c** – HT vs. FCA; **d** – NG vs. control; **e** – NG vs. FCA; **f** – PTC vs. FCA; **g** – PTC vs. NG; **h** – HT vs. NG; **i** – FCA vs. control.


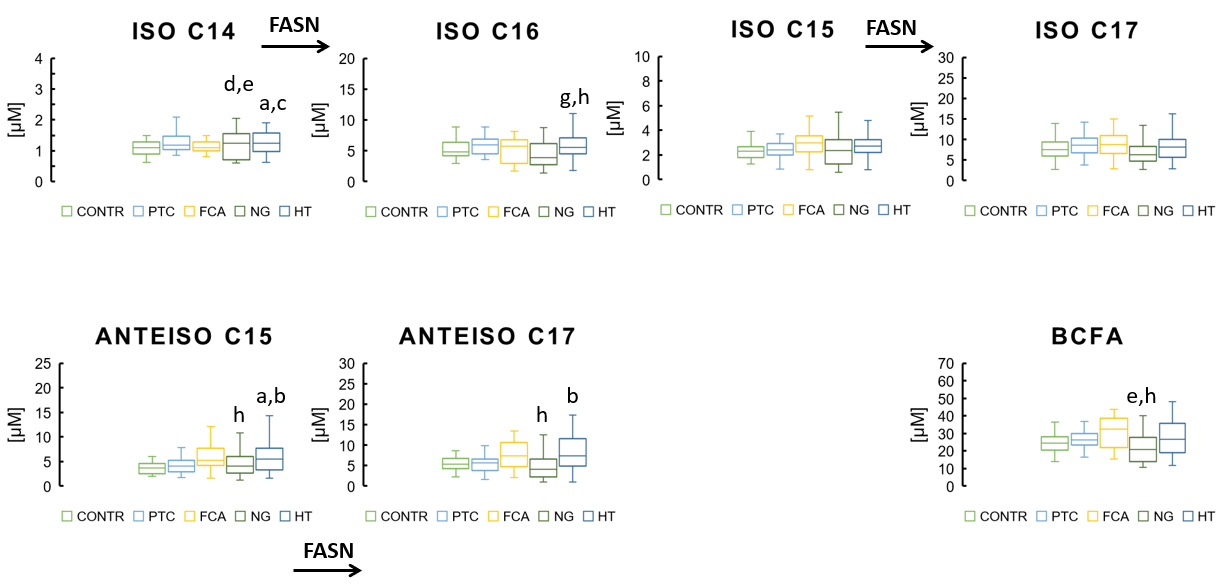


**Figure S9:** Branched-chain fatty acid concentrations in study subjects’ serum. FASN - fatty acid synthase. **a** – HT vs. control; **b** - HT vs. PTC; **c** – HT vs. FCA; **d** – NG vs. control; **e** – NG vs. FCA; **f** – PTC vs. FCA; **g** – PTC vs. NG; **h** – HT vs. NG, **i** – FCA vs. control.


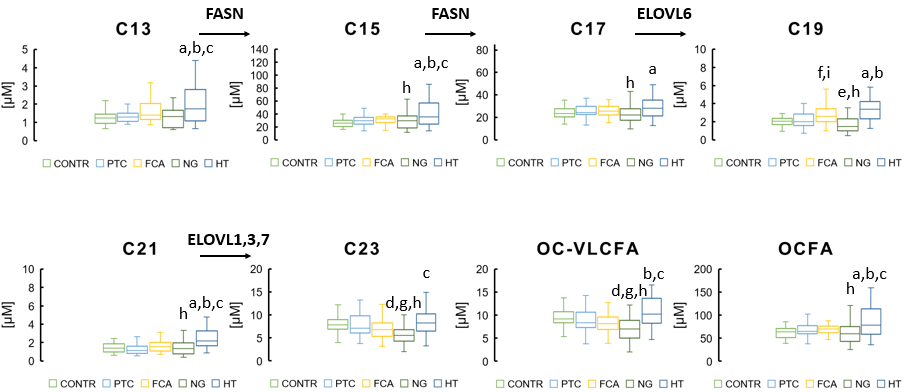


**Figure S10:** Odd-chain fatty acid concentrations in study subjects’ serum. OC-VLCFA – odd-chain very long-chain FAs, FASN - fatty acid synthase, ELOVL1,3,7 - fatty acid elongase 1,3,7. **a** – HT vs. control; **b** - HT vs. PTC; **c** – HT vs. FCA; **d** – NG vs. control; **e** – NG vs. FCA; **f** – PTC vs. FCA; **g** – PTC vs. NG; **h** – HT vs. NG; **i** – FCA vs. control.


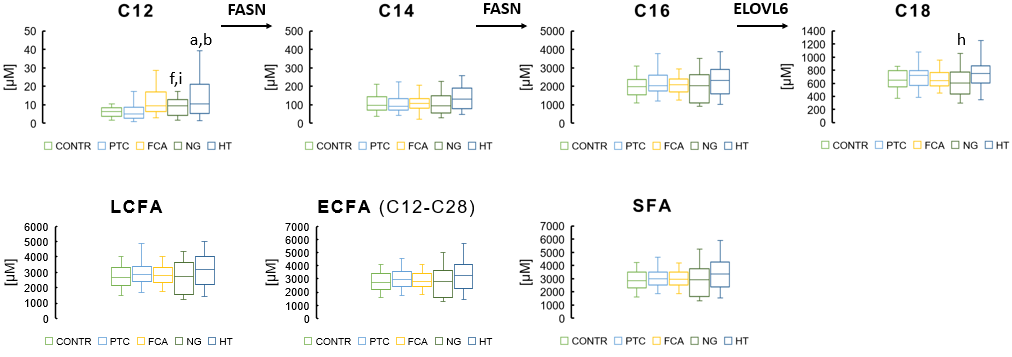


**Figure S11:** Saturated fatty acid concentrations in study subjects’ serum. LCFA – long-chain FAs, ECFA – even-chain FAs, SFA – saturated fatty acids, FASN - fatty acid synthase, ELOVL6 - fatty acid elongase 6. **a** – HT vs. control; **b** - HT vs. PTC; **c** – HT vs. FCA; **d** – NG vs. control; **e** – NG vs. FCA; **f** – PTC vs. FCA; **g** – PTC vs. NG; **h** – HT vs. NG; **i** – FCA vs. control.


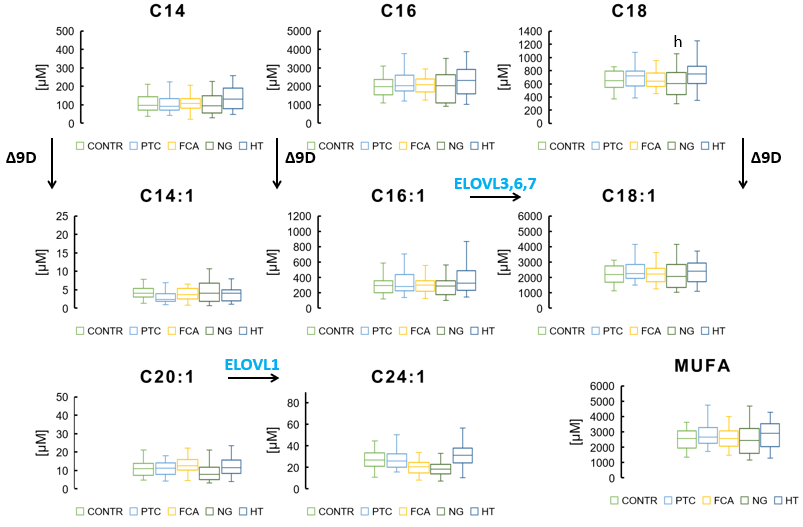


**Figure S12:** Saturated and monounsaturated fatty acids concentrations in study subjects’ serum. MUFA - monounsaturated FAs, Δ9D - Δ9 fatty acid desaturase, ELOVL1,3,6,7 - fatty acid elongase 1,3,6,7. **a** - HT vs. control; **b** - HT vs. PTC; **c** – HT vs. FCA; **d** – NG vs. control; **e** – NG vs. FCA; **f** – PTC vs. FCA; **g** – PTC vs. NG; **h** – HT vs. NG; **i** – FCA vs. control.

Supplementary Table 4. Classical ROC curve analysis for individual biomarkers when the target group is PTC.

| **Name** | **AUC** | **T-test** | **Log2fc** |
| --- | --- | --- | --- |
| lauric acid | 0.69117 | 0.0018476 | -0.84669 |
| α-linolenic acid | 0.68045 | 0.0015853 | 0.49065 |
| heneicosanoic acid | 0.66626 | 0.0031784 | -0.48232 |
| docosapentaenoic acid n-3 | 0.66316 | 0.0010755 | 0.35019 |
| arachidonic acid | 0.66071 | 0.005779 | 0.26442 |
| polyunsaturated fatty acid n-3 | 0.66015 | 0.015397 | 0.31876 |
| myristoleic acid | 0.65423 | 0.0085233 | -0.47981 |
| docosahexaenoic acid | 0.64756 | 0.061333 | 0.2514 |
| dihomo-gamma-linolenic acid | 0.64455 | 0.0062308 | 0.26988 |
| lignoceric acid | 0.6344 | 0.081448 | -0.11844 |
| tridecanoic acid | 0.6328 | 0.0080659 | -0.45576 |
| very long-chain fatty acids | 0.63083 | 0.28008 | -0.072649 |
| adrenic acid | 0.62998 | 0.021363 | 0.26928 |
| iso C16 | 0.62509 | 0.054726 | 0.20963 |
| behenic acid | 0.6235 | 0.21282 | -0.097459 |
| nervonic acid | 0.61861 | 0.062059 | 0.16421 |
| eicosatetraenoic acid | 0.61617 | 0.068003 | 0.23707 |
| docosapentaenoic acid n-6 | 0.61588 | 0.002738 | 0.37792 |
| polyunsaturated fatty acid n6 | 0.61335 | 0.11453 | 0.13101 |
| polyunsaturated fatty acids | 0.60949 | 0.18295 | 0.15043 |
| eicosapentaenoic acid | 0.59991 | 0.41058 | 0.20249 |
| eicosadienoic acid | 0.5968 | 0.07415 | 0.1792 |
| cerotic acid | 0.59352 | 0.031986 | -0.39719 |
| anteiso branched-chain fatty acids | 0.59126 | 0.032203 | -0.26637 |
| anteiso C17 | 0.58393 | 0.040927 | -0.33573 |
| myristic acid | 0.57951 | 0.25868 | -0.15849 |
| anteiso C15 | 0.57434 | 0.081599 | -0.26348 |
| iso branched-chain fatty acids | 0.57434 | 0.12559 | 0.13769 |
| heptadecanoic acid | 0.56767 | 0.50168 | 0.056768 |
| nonadecanoic acid | 0.56579 | 0.090324 | -0.19153 |
| tricosanoic acid | 0.56429 | 0.22502 | 0.11082 |
| oleic acid | 0.56034 | 0.31412 | 0.096963 |
| iso C15 | 0.55648 | 0.12984 | -0.15987 |
| arachidic acid | 0.55508 | 0.1981 | -0.1295 |
| linoleic acid | 0.55188 | 0.53676 | 0.063487 |
| monounsaturated fatty acids | 0.55113 | 0.40025 | 0.085237 |
| iso C17 | 0.55009 | 0.55744 | 0.061709 |
| palmitic acid | 0.54887 | 0.53122 | 0.072237 |
| stearic acid | 0.54192 | 0.93568 | 0.029267 |
| even-chain fatty acids | 0.54154 | 0.60813 | 0.062796 |
| long-chain fatty acids | 0.53872 | 0.69074 | 0.050772 |
| nonadecenoic acid | 0.52914 | 0.2713 | -0.18046 |
| palmitoleic acid | 0.52472 | 0.69165 | 0.072497 |
| branched-chain fatty acids | 0.52246 | 0.7588 | 0.047397 |
| iso C14 | 0.51974 | 0.75365 | 0.10273 |
| pentadecanoic acid | 0.51739 | 0.6237 | -0.064255 |
| odd-chain fatty acids | 0.51335 | 0.952 | 0.0046954 |
| saturated fatty acids | 0.50921 | 0.35567 | -0.060922 |
| odd chain very long chain fatty acids | 0.50771 | 0.90454 | 0.02403 |
| eicosenoic acid | 0.50733 | 0.91919 | 0.025366 |

The features displayed in the table below are ranked based on the area under ROC curve (AUROC), T-statistics and Log2 fold change (FC). The 95% confidence interval is calculated using 500 boot strappings. Additional parameters: threshold 0.2 and optimal cutoff using closest to top-left corner.
